# The more we search, the more we find: discovering and expanding the biodiversity in the ring nematode genus *Xenocriconemella* De Grisse and Loof, 1965 (Nematoda: Criconematidae)

**DOI:** 10.1186/s40851-024-00230-3

**Published:** 2024-03-25

**Authors:** A. Archidona-Yuste, I. Clavero-Camacho, A. N. Ruiz-Cuenca, C. Cantalapiedra-Navarrete, G. Liebanas, P. Castillo, J. E. Palomares-Rius

**Affiliations:** 1 Department of Crop Protection, Institute for Sustainable Agriculture (IAS), Spanish National Research Council (CSIC), Avda. Menendez Pidal s/n, Campus de Excelencia Internacional Agroalimentario, ceiA3, 14004 Córdoba, Spain; 2 Department of Animal, Plant Biology and Ecology, University of Jaén, Campus Las Lagunillas, Jaén, Spain

**Keywords:** Species complex, Integrative taxonomy, Morphometry, Ribosomal and mitochondrial markers

## Abstract

The ring nematode genus *Xenocriconemella* De Grisse and Loof, 1965 comprises only one nominal species, *Xenocriconemella macrodora* (Taylor, 1936) De Grisse and Loof, 1965. The initial objective of the present study was to investigate the morphological–morphometric and molecular diversity of 28 *X. macrodora* populations in the Iberian Peninsula associated with tree forests (mainly *Quercus* spp.). However, a detailed integrative taxonomic analysis (morphological–morphometric and molecular data) from each population and analysis of this data using principal component analysis (PCA) for morphometric data (including these 28 populations and other 25 *X. macrodora* populations around the world) and molecular and phylogenetic species delimitation methods revealed that *X. macrodora* forms a species complex. This species complex is composed by species that are morphometricly and morphologically similar, but clearly different at the molecular level. Three new species are described applying integrative taxonomy, namely as *Xenocriconemella iberica* sp. nov., *Xenocriconemella paraiberica* sp. nov. and *Xenocriconemella pradense* sp. nov. However, the molecular diversity of this species in USA and Italy confirmed that additional species are likely present in this species complex, and the diversity of this group may be higher than expected. The study of *X. macrodora* topotypes can clarify the position of this species using molecular markers under an integrative approach.

**Supplementary Information:**

The online version contains supplementary material available at 10.1186/s40851-024-00230-3.

## Introduction

The ring nematode genus *Xenocriconemella* De Grisse and Loof, 1965 [1] comprises obligate ectoparasite nematodes included under subfamily Discocriconemellinae Geraert, 2010 [2] and is characterized by a body length *ca*. 250–300 µm, a long and flexible stylet (*ca*. 100–140 µm and up to 40% of body length), lip region annulated but lacking submedian lobes or pseudolips, body annuli smooth without anastomosis, vulva closed, and juveniles similar to females [2]. *Xenocriconemella macrodora* (Taylor, 1936) De Grisse and Loof, 1965 [1, 3] is the only nominal species within the genus.

**Fig. 1:**
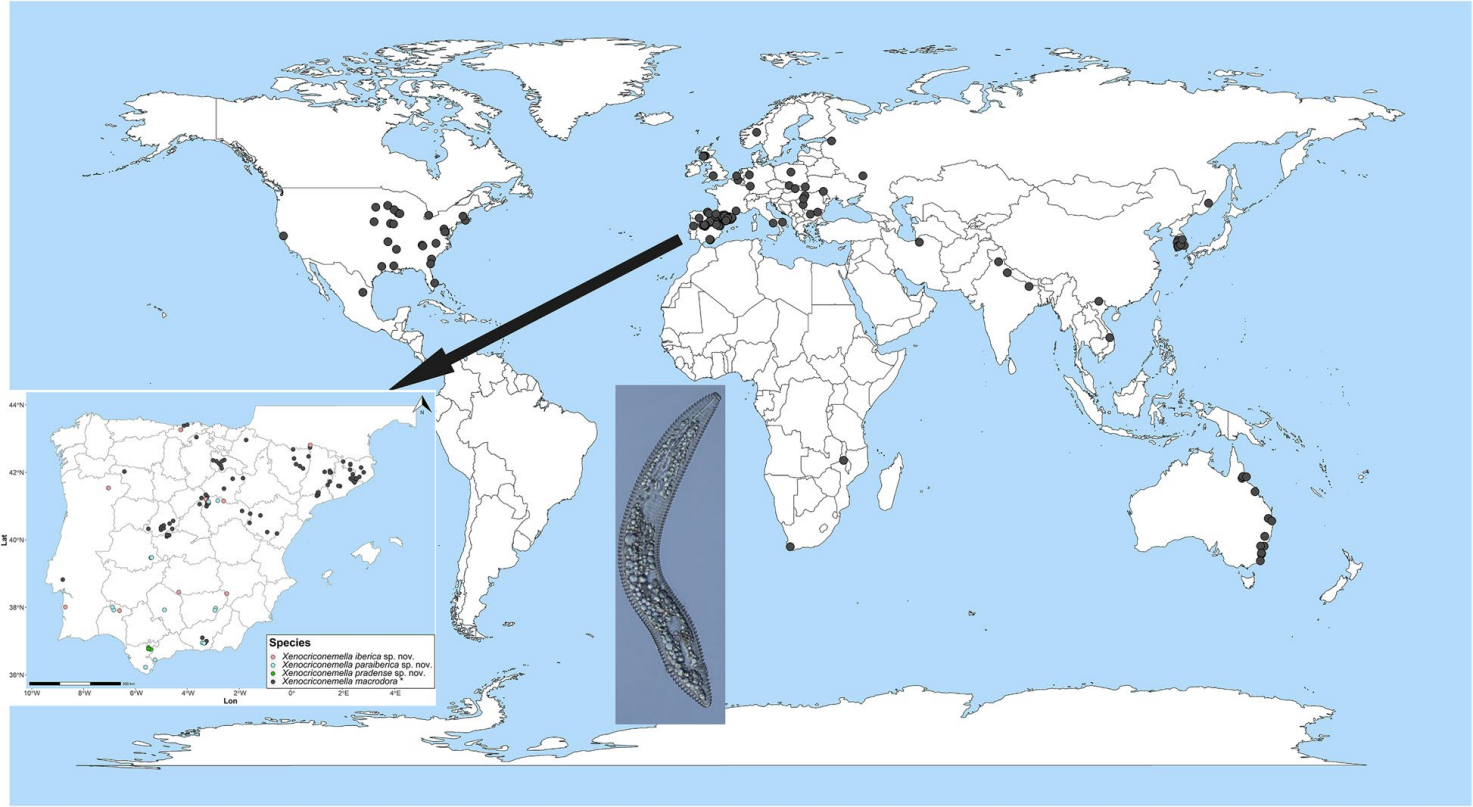
Global distribution of the ring nematode *Xenocriconemella macrodora* De Grisse and Loof, 1965, highlighting its distribution in the Iberian Peninsula (black circles) and the 28 sampling points studied here, indicating the three new species described herein in coloured circles

A world review of *X. macrodora* indicated that this species occurs in association with woodland forests [4, 5]. This species is distributed worldwide (Fig. 1), with a widespread presence in USA [5] and several European countries, particularly in Spain [4, 6]. Owing to its large morphological diversity, some taxonomic studies have challenged the possibility that the genus *Xenocriconemella* is monospecific. Lϋbbers and Zell [7] morphologically studied several populations of *X. macrodora* from Germany and concluded that German populations differed morphologically from those of the USA and several European countries in a higher number of annuli (R = 147 *vs* R = 99-120), concluding that these populations belong to a new species, *X. degrissei* Lϋbbers and Zell, 1989. Later on, Ganguly et al. [8] also studied *X. macrodora* populations from peach and blue pine in India and concluded that these populations belong to two new species, *X. pruni* Ganguly et al. 2008 and *X. pini* Ganguly et al. 2008. However, Sturhan [9] compared the morphometry of all three species and concluded that all three species overlap with populations of *X. macrodora*, disallowing the existence of these proposed new taxa, but no molecular markers were provided to confirm this action. Only ribosomal and mitochondrial sequences are available in NCBI from the USA [5, 10] and Italy [11], despite its being a cosmopolitan species, which may be recognized as a major gap in nematode biodiversity knowledge. Molecular taxonomy and DNA barcoding can provide definitively accurate and useful tools for assessing populations and species boundaries in the genus *Xenocriconemella* only through integrative-based taxonomy studies (combination of morphology-morphometry with molecular data) as in other *Criconematidae* spp. [5, 10–15].

The initial objective of the present study was to investigate the morphological–morphometric and molecular diversity of *X. macrodora* populations in the Iberian Peninsula associated with tree forests (mainly *Quercus* spp.) (Fig. 1), and compared with the available molecular cytochrome c oxidase subunit 1 (COI) barcodes of *X. macrodora* from the USA [5] and ribosomal sequences from Italy [11]. However, the scarce similarity values detected among mitochondrial and ribosomal sequences from the Iberian Peninsula populations and the available accessions from USA and Italy populations prompted us to carry out detailed morphological, morphometric and molecular studies on these populations to clarify the taxonomic status of these ring nematode populations, thus determining whether it is a new case of cryptic species complex within the genus *Xenocriconemella*, such as those recently described in the genera *Mesocriconema* [16], *Criconemoides* [17], or *Criconema* [15, 18]. Notably, we will follow the classification proposed by Geraert [2], representing the widely accepted classification of this group of nematodes [14] and separating *Criconemoides* from *Xenocriconemella*. Hence, the main objectives of this study were to (i) accurately identify with morphological and morphometric approaches several populations of *Xenocriconemella* detected in an extensive nematode survey in natural habitats in the Iberian Peninsula, (ii) discover the diversity of *Xenocriconemella* populations through integrative taxonomy, combining morphological analysis and a species delineation approach based on multivariate analysis of morphometric data and genetic methods; (iii) describe three new species of the genus *Xenocriconemella* belonging to the *X. macrodora* species complex; (iv) provide molecular characterization of these *Xenocriconemella* populations using ribosomal (D2-D3 expansion segments of 28S rRNA, ITS region, partial 18S rRNA) and COI markers; and (v) study phylogenetic relationships within *Criconematidae* spp. and these species of the *X. macrodora* species complex.

## Materials and methods

### Sampling

A nematological survey was conducted in the principal areas and sampling sites where *X. macrodora* had been reported in the Iberian Peninsula [4, 6, 19], as well as new areas not previously sampled in Spain and Portugal. Since *X. macrodora* has been associated with oak forests [4, 5], most of the sampling program was concentrated on *Quercus* spp. forests (including *Quercus canariensis* Willd., *Quercus faginea* Lam., *Quercus ilex* L., *Quercus pyrenaica* Willd., *Quercus pubescens* Willd., *Quercus suber* L.), as well as *Castanea sativa* Mill. and *Fagus sylvatica* L., covering the majority of the area of the potential distribution of *X. macrodora* in the Iberian Peninsula from south to north (Table 1, Fig. 1). A total of 28 sites showed the presence of specimens of putative *X. macrodora*. An additional soil sample was collected in the type locality of the ring nematode *Criconemoides rosmarini* (Castillo, Siddiqi and Gómez-Barcina, 1988) [20] Siddiqi, 2000 to molecularly characterize and check the phylogenetic relationships with *Xenocriconemella*. Soil samples for nematode analysis were collected with a shovel from two randomly selected trees and mixed to constitute a soil sample from each sampling site; samples came from the upper 5–40 cm depth of soil. Nematodes were extracted by centrifugal flotation from a 500 cm^3^ soil subsample [21].

**Table 1:** Host-plant species and localities of the analysed populations of the *Xenocriconemella* De Grisse & Loof, 1965 species complex from Spain in this study

**Nematode species**		**Host-plant species**	**Locality, province, Country**	**Abundance**	**NCBI Accessions**			
	**Code**			**(Nem/500 cm**^**3**^** soil)**	**D2-D3**	**ITS**	**18S**	**COI**
*Xenocriconemella iberica* sp. nov.	COT22	*Quercus pyrenaica* Willd.	Cotillas, Albacete, Spain (type)	1660	OR880107-OR880112	OR878332-OR878334	OR878356-OR878357	OR885933-OR885938
*Xenocriconemella iberica* sp. nov.	HUC01	*Castanea sativa* Mill.	Aracena, Huelva, Spain	2008	OR880123-OR880128	-	-	OR885948-OR88595
*Xenocriconemella iberica* sp. nov.	PIR17	*Quercus pubescens* Willd.	Les, Lleida, Spain	38	OR880113-OR880117	OR878335-OR878337	-	OR885939-OR885942
*Xenocriconemella iberica* sp. nov.	FCQ02	*Quercus faginea* Lam.	Fuencaliente; Ciudad Real, Spain	74	OR880118-OR880121	-	-	OR885943-OR885946
*Xenocriconemella iberica* sp. nov.	GUR05	*Quercus pyrenaica* Willd.	Guadalupe, Cáceres, Spain	176	OR880122	-	-	OR885947
*Xenocriconemella iberica* sp. nov.	PTA01	*Quercus suber* L.	Santiago do Cacem, Estremadura, Portugal	39	OR880129-OR880133	-	-	OR885954-OR885958
*Xenocriconemella iberica* sp. nov.	PTQ01	*Quercus canariensis* Willd.	Mirandela, Tras-os-Montes, Portugal	10	OR880134-OR880137	-	-	OR885959-OR885961
*Xenocriconemella iberica* sp. nov.	PTA02	*Quercus suber* L.	T. Moncorvo, Tras-os-Montes, Portugal	3	OR880138-OR880139	-	-	OR885962-OR885963
*Xenocriconemella iberica* sp. nov.	XEN37	*Quercus ilex* L.	Alboreca, Guadalajara, Spain	160	OR880140-OR880142	-	-	OR885964-OR885965
*Xenocriconemella iberica* sp. nov.	XN48B	*Quercus pyrenaica* Willd.	Cantalojas, Guadalajara, Spain	66	OR880143-OR880144	-	-	OR885966-OR885968
*Xenocriconemella iberica* sp. nov.	XN56B	*Quercus pyrenaica* Willd.	Umbralejo, Guadalajara, Spain	34	OR880145-OR880146	-	-	OR885969-OR885971
*Xenocriconemella iberica* sp. nov.	HCANT	*Fagus sylvatica* L.	Ucieda, Santander, Spain	19	OR880147-OR880148	-	-	OR885972-OR885977
*Xenocriconemella iberica* sp. nov.	RCANT	*Quercus pyrenaica* Willd.	Gismana, Santander, Spain	240	OR880149-OR880151	-	-	OR885978-OR885982
*Xenocriconemella paraiberica* sp. nov.	CAS22	*Quercus suber* L.	Casares, Málaga, Spain (type)	2310	OR880152-OR880161	OR878338-OR878342	OR878358-OR878359	OR885983-OR885987
*Xenocriconemella paraiberica* sp. nov.	CZQ05	*Quercus faginea* Lam.	Arroyo Frío, Jaén, Spain	44	OR880162-OR880164	OR878343-OR878345	-	OR885988-OR885991
*Xenocriconemella paraiberica* sp. nov.	COA01	*Quercus suber* L.	Trassierra, Córdoba, Spain	65	OR880165-OR880169	OR878346-OR878349	-	OR885992-OR885993
*Xenocriconemella paraiberica* sp. nov.	CAC01	*Quercus canariensis* Willd.	Los Barrios, Cádiz, Spain	59	OR880170-OR880175	-	-	OR885994-OR885996
*Xenocriconemella paraiberica* sp. nov.	CNR03	*Quercus pyrenaica* Willd.	Cañar, Granada, Spain	244	OR880176-OR880179	-	-	OR885997-OR886000
*Xenocriconemella paraiberica* sp. nov.	HUE00	*Quercus ilex* L.	Cortegana, Huelva, Spain	11	OR880180	-	-	OR886001
*Xenocriconemella paraiberica* sp. nov.	HUA03	*Quercus suber* L.	Aroche, Huelva, Spain	13	OR880181-OR880182	-	-	OR886002-OR886004
*Xenocriconemella paraiberica* sp. nov.	BUQ01	*Quercus faginea* Lam.	Bubión, Granada, Spain	5	OR880183-OR880186	-	-	OR886005
*Xenocriconemella paraiberica* sp. nov.	GUR04	*Quercus pyrenaica* Willd.	Guadalupe, Cáceres, Spain	920	OR880187-OR880191	-	-	OR886006-OR886010
*Xenocriconemella paraiberica* sp. nov.	GUR03	*Quercus pyrenaica* Willd.	Guadalupe, Cáceres, Spain	103	OR880192-OR880196	-	-	OR886011-OR886014
*Xenocriconemella paraiberica* sp. nov.	XN43A	*Quercus pyrenaica* Willd.	Atienza, Guadalajara, Spain	14	OR880197-OR880199	-	-	OR886015-OR886017
*Xenocriconemella paraiberica* sp. nov.	XN55A	*Quercus pyrenaica Willd.*	Umbralejo, Guadalajara, Spain	258	OR880200-OR880202	-	-	OR886018-OR886019
*Xenocriconemella pradense* sp. nov.	GRQ01	*Quercus faginea* Lam.	Prado del Rey, Cádiz, Spain (type)	139	OR880203-OR880208	OR878350-OR878351	OR878360-OR878361	OR886020-OR886023
*Xenocriconemella pradense* sp. nov.	GRQ02	*Quercus faginea* Lam.	Prado del Rey, Cádiz, Spain	224	OR880209-OR880213	OR878352-OR878353	-	OR886024-OR886026
*Xenocriconemella pradense* sp. nov.	GRQ05	*Quercus faginea* Lam.	Prado del Rey, Cádiz, Spain	612	OR880214-OR880218	OR878354-OR878355	-	OR886027-OR886030

### Morphology

Nematode identification was completed using an integrative approach, combining morphological and morphometric evaluation with molecular techniques. Morphological and morphometric analyses were conducted using fixed individuals mounted on permanent slides. To prepare the fixed material, specimens of *Xenocriconemella* specimens were killed at 70–75 °C and fixed in an aqueous solution of 4% formaldehyde + 1% glycerol, dehydrated using an alcohol-saturated chamber and processed to pure glycerin using Seinhorst’s method [22] as modified by De Grisse [23]. A total of 185 individuals, including 183 females and two males were used for morphological and morphometric analyses. Fixed, mounted individuals were then examined, and measurements of each nematode population were performed using a Leica DM6 compound microscope with a Leica DFC7000 T digital camera. Morphological terminologies follow Archidona-Yuste et al. [18]. Measurements and ratios included: n, number of specimens studied; L, (total body length); a = body length/maximal body width; b = body length/pharyngeal length; c = body length/tail length; c’ = tail length/body width at anus; O = distance between stylet base and orifice of dorsal pharyngeal gland as percentage of stylet length; *R* = total number of body annuli; Roes = number of annuli in pharyngeal region; Rex = number of annuli between anterior end of body and excretory pore; Rst = number of body annuli between labial disc and stylet knobs; RV = number of annuli between posterior end of body and vulva; Rvan = number of annuli between vulva and anus; Ran = number of annuli on tail; V = (distance from anterior end to vulva/body length) × 100; VL/VB = distance between vulva and posterior end of body divided by body width at vulva; T = (distance from cloacal aperture to anterior end of testis/body length) × 100 [18].

Females of each species mounted in glycerin were selected for SEM observations. The nematodes were hydrated in distilled water, dehydrated in a graded ethanol-acetone series, critical point-dried, coated with gold, and observed with a Zeiss Merlin scanning electron microscope (5 kV) (Zeiss, Oberkochen, Germany) [24].

### DNA extraction, PCR and Sequencing

For molecular analyses, and to avoid mistakes in the case of mixed populations in the same sample, single nematodes were pre-mounted in a drop of NaCl and used for molecular identification after recording morphological data. Genomic DNA extraction from single specimens was conducted as described by Archidona-Yuste et al. [18]. Briefly, an individual nematode was cut using a scalpel in a drop of PCR buffer (ThermoPol®, Biolabs, New England, USA) (20 μL), and 2 μL proteinase K (600 μg/mL) was added. Tubes were frozen at −80°C (15 min) and then incubated at 65°C (1 h) and 95°C (10 min) consecutively. Tubes were centrifuged (1 min, 16,000 × *g*) and kept at −20°C until use in PCR; more importantly, all three molecular markers for each population of *Xenocriconemella* were extracted from the same single individual in each PCR tube without any exception. In addition, male conspecificity was confirmed by single DNA extraction of males.

The D2-D3 expansion domains of the 28S rRNA were amplified using the D2A (5′-ACAAGTACCGTGAGGGAAAGTTG-3′) and D3B (5′-TCGGAAGGAACCAGCTACTA-3′) primers [25]. The ITS region was amplified by using forward primer TW81 (5′-GTTTCCGTAGGTGAACCTGC-3′) and reverse primer AB28 (5′-ATATGCTTAAGTTCAGCGGGT-3′) [26]. The partial 18S rRNA was amplified using the primers 988 (5′-CTCAAAGATTAAGCCATGC-3′), 1912R (5′-TTTACGGTCAGAACTAGGG-3′), 1813F (5´- CTGCGTGAGAGGTGAAAT -3´), and 2646R (5´- GCTACCTTGTTACGACTTTT -3´) [27]. The COI gene was amplified using the primers JB3 (5´- TTTTTTGGGCATCCTGAGGTTTAT-3`) and JB5 (5’- AGCACCTAAACTTAAAACATAATGAAAATG -3´) [28, 29]. The PCR cycling conditions for the 28S rRNA, ITS and 18S rRNA were as follows: 95°C for 15 min, followed by 35 cycles of 94°C for 30 s, an annealing temperature of 55°C for 45 s, and 72°C for 1 min, and one final cycle of 72°C for 10 min. The PCR cycling for COI primers was as follows: 95°C for 15 min, 39 cycles at 94°C for 30 s, 53°C for 30 s, and 68°C for 1 min, followed by a final extension at 72°C for 7 min. The PCR volumes were adapted to 20 μL for each reaction, and primer concentrations were as described in De Ley et al. [25], Subbotin et al. [11], Holterman et al. [27] and Powers et al. [30]. We used 5x HOT FIREpol Blend Master Mix (Solis Biodyne, Tartu, Estonia) in all PCRs. The PCR products were purified using ExoSAP-IT (Affimetrix, USB products, Kandel, Germany) and used for direct sequencing in both directions with the corresponding primers. The resulting products were analysed in a DNA multi-capillary sequencer (Model 3130XL Genetic Analyzer; Applied Biosystems, Foster City, CA, USA), using the BigDye Terminator Sequencing Kit v.3.1 (Applied Bio-systems) at the Stab Vida sequencing facility (Caparica, Portugal). The sequence chromatograms of the four markers (18S rRNA, ITS, COI and D2-D3 expansion segments of 28S rRNA) were analysed using DNASTAR LASERGENE SeqMan v. 7.1.0. The Basic Local Alignment Search Tool (BLAST) at the National Center for Biotechnology Information (NCBI) was used to confirm the species identity of the DNA sequences obtained in this study [31]. The newly obtained sequences were deposited in the GenBank database under accession numbers indicated on the phylogenetic trees and in Table 1.

### Species delimitation within the *Xenocriconemella macrodora* species complex

Two independent strategies of species delimitation were used to determine species boundaries within the *X. macrodora* species complex including morphometric and molecular data.

Species delineation using morphometry was conducted using principal component analysis (PCA) [32]. We established the species delimitation among these morphometrically similar new unresolved populations of *Xenocriconemella* found in the Iberian Peninsula, and we further assessed the relationships between these new populations with those already described as *X. macrodora*. PCA was based upon the following morphological characters: L, stylet length, R, Rst, Roes, Rex, RV, Rvan, Ran, and the ratios a, b, c, V, VL/VB [18]. Depending on data availability, we selected 25 *X. macrodora* populations previously reported from several countries for comparison with the 28 new Iberian populations of *X. macrodora* studied under an integrative taxonomical approach (Table 1 and S1). In the previously reported data, we used the average values of the morphological characters mentioned above in each population. Diagnostic characters were previously standardized and tested for collinearity [33]. We used the collinearity test based on the values of the variance inflation factor (VIF) method that iteratively excludes numeric covariates showing VIF values > 10 as suggested by Montgomery et al. [34]. PCA was performed using the PCA function implemented in the software package ‘FactoMineR’ [35]. All data analyses were done with the R version 4.2.2 (R Core Team, 2022; https://www.R-project.org).

Species delineation based on molecular data was performed using the species delimitation plugin [36] from the program Geneious Prime v2022.1.1. (Geneious, Auckland, New Zealand), and was used to calculate intra- and inter-species variation by means of the P ID liberal and Rosenberg’s P_AB_ value. The intra- and inter-species molecular variation was determined by calculating the ratio between the average genetic distance between individuals within a species and the average genetic distance between individuals belonging to the sister species [36]. The P ID (Liberal) value [37] represents the probability that a correct species identification would be made using the best sequence alignment (BLAST), closest genetic distance or placement on a tree (falling within or being sister to a monophyletic species clade). Species with P ID (Liberal) ≥ 0.93 were considered to be adequately delimited [38]. Rosenberg’s P_AB_ represents the probability that the monophyly of a group of sequences is the result of random branching [39].

### Phylogenetic analyses

The D2-D3 expansion segments of the 28S rRNA, ITS rRNA, 18S rRNA, and COI mtDNA sequences of the 28 populations of *Xenocriconemella* were obtained in this study. These sequences and other sequences of Criconematidae spp. from GenBank were used for phylogenetic analyses. The selection of outgroup taxa for each dataset was based on previously published studies [13, 14, 40]. Multiple sequence alignments of the different genes were completed using the FFT-NS-2 algorithm of MAFFT V.7.450 [41]. The BioEdit program V. 7.2.5 [42] was used for sequence alignment visualization and manually edited and trimmed of the poorly aligned positions using a light filtering strategy (up to 20% of alignment positions), which has little impact on tree accuracy and may save computation time, as suggested by Tan et al. [43], since methods for automated filtering of multiple sequence alignments frequently worsen single-gene phylogenetic inference [43]. Phylogenetic analyses of the sequence datasets were based on Bayesian inference (BI) using MrBayes 3.1.2 [44]. The best-fit model of DNA evolution was achieved using JModelTest V.2.1.7 [45] with the Akaike information criterion (AIC). The best-fit model, the base frequency, the proportion of invariable sites, and the gamma distribution shape parameters and substitution rates in the AIC were then used in MrBayes for phylogenetic analyses. The general time-reversible model with invariable sites and a gamma-shaped distribution (GTR + I + G) for the D2-D3 segments of 28S rRNA, the transversion model with invariable sites and a gamma-shaped distribution (TVM + I + G) for the ITS rRNA region, and the transition models with invariable sites and a gamma-shaped distribution (TIM2ef + I + G, TIM3 + I + G) for the partial 18S rRNA gene and COI gene were run with four chains for 4 × 10^6^ generations. A combined analysis of the three ribosomal genes was not undertaken because some sequences were not available for all species. The sampling for Markov chains was conducted at intervals of 100 generations. For each analysis, two runs were conducted. After discarding burn-in samples of 30% and evaluating convergence, the remaining samples were retained for more in-depth analyses. The topologies were used to generate a 50% majority-rule consensus tree. For each appropriate clade, posterior probabilities (PP) were given. FigTree software version v.1.4.3 [46] was used for visualizing trees from all analyses.

The alignment for COI sequences was used to determine haplotypes of the COI gene using DnaSP5 software [47], while the haplotype network map of all haplotypes of the COI gene for each species was constructed using the TCS network of PopART V. 1. 7. (Population Analysis with Reticulate Trees) software (http://popart.otago.ac.nz) [48].

## Results

The 28 ring nematode populations clearly resembling *Xenocriconemella macrodora* were mainly associated with *Quercus* spp., but also with *Castanea sativa* and *Fagus sylvatica* from several natural environments in the Iberian Peninsula (Portugal and Spain) (Table 1). Nematode populations showed a mean density of 361 nematodes/500 cm^3^ of soil, but ranged from very low (three nematodes/500 cm^3^ of soil) to very high (2310 nematodes/500 cm^3^ of soil) soil nematode densities in a sample from cork oak in northern Portugal and a sample from cork oak in southern Spain (Table 1). All these populations were identified herein using integrative taxonomical approaches (morphometric and molecular) and a new species complex within the morphospecies *X. macrodora* was described herein separating three new species of *Xenocriconemella*. These 28 populations were separated into 13 populations of *X. iberica* sp. nov., 12 populations of *X. paraiberica* sp. nov., and 3 populations of *X. pradense* sp. nov. based on different approaches explained below.

### Species delimitation using morphometry

**Fig. 2:**
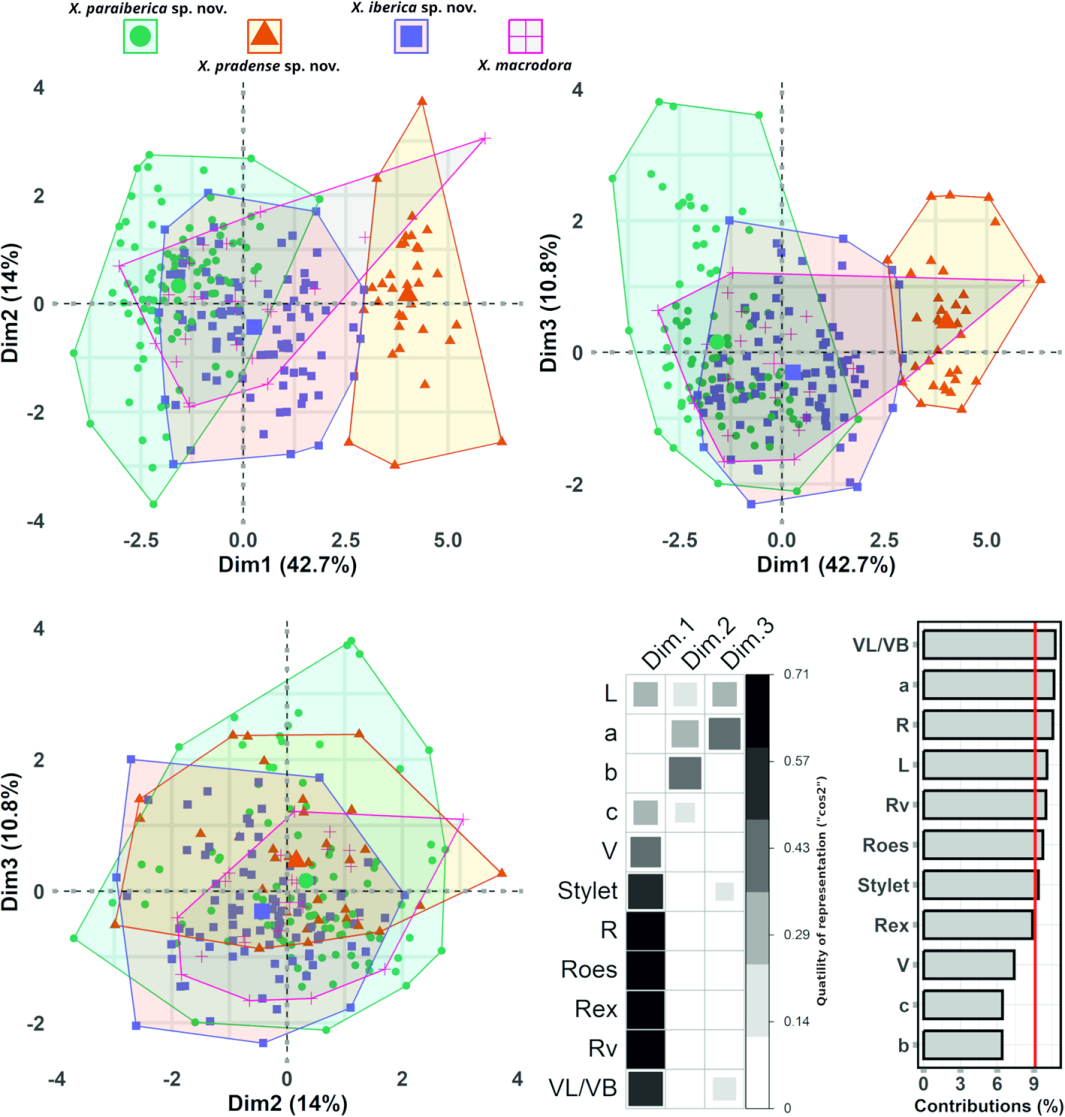
Principal component on *Xenocriconemella macrodora* species complex. Projections of species on the plane of dimensions 1 and 2, 1 and 3, 2 and 3. Correlation plot between dimensions and qualities of representation of the morphometric characters (“square cosine” (cos2)). Barplot showing the standardized contribution (%) of morphometric variables for the three dimensions retained by the PCA (only dimensions with sum of squares (SS) loadings > 1 were extracted). A reference soil (red) line is also shown on the barplot. This reference line corresponds to the expected value if the contribution where uniform. For a given dimension, any row/column with a contribution above the reference line can be considered important in contributing to the dimension

In PCA results, the first three components (sum of squares (SS) loadings>1) accounted for 68% of the total variance in the morphometric characteristics when considering new and described taxa within the genus *Xenocriconemella*. Notably, our results showed significant results when the dimensional reductions were plotted (Fig. 2). First, we observed a wide intraspecific variation among the specimens in each *Xenocriconemella* spp. based on the wide morphometric variation in stylet and body length and width (i.e. L, a and Stylet), body annuli feature (i.e. R, Rv, Roes and Rex) and the distance between vulva and posterior end of body divided by body width at vulva position (VL/VB) (see contributions in Fig. 2). As expected, our findings supported the notable morphological diversity exhibited by *X. macrodora* (Table S1). PCA clearly separated almost all specimens of *X. pradense* sp. nov from those belonging to *X. iberica* sp. nov. and *X. paraiberica* sp. nov. However, it should be noted that this spatial separation was to a lesser extent between *X. pradense* sp. nov and *X. iberica* sp. nov., where several specimens were located close to each other (Fig. 2). This species separation was mostly observed along the dimension (Dim 1; 42.7% of the total variance). Considering that the eigenvalues for each character were used to identify the key morphological characters for this species delimitation (see the quality of representation of the variables in Fig. 2), the Dim 1 was mainly dominated by the body annuli feature (i.e. R, Rv, Roes and Rex) and subsequently by stylet length, V and VL/VB. We mainly detected that species separations were based in the number of annuli in body (R), in the in pharyngeal region (Roes), and between posterior end of body and vulva (Rv). More specifically, specimens with higher values in R, Roes and Rv were located on the right (i.e. *X. pradense* sp. nov.), and those with lower values for these traits on the left side of the dimension 1 (i.e. *X. paraiberica* sp. nov.). On the contrary, most of the specimens of *X. iberica* sp. nov. and *X. paraiberica* sp. nov. were found overlapping each other because they showed similar values for characters associated with Dim 1 (Fig. 2; see species description section). This therefore implied that both species are closely related morphologically. PCA showed a similar pattern in the spatial distribution of specimens (i.e., mean values of populations) of *X. macrodora*. In this case, the populations of the species already described were located throughout the spatial projection occupied by each of the new species given the wide morphometric variation that describes the populations of *X. macrodora* (Fig. 2, Table S1). This result supports the idea that this already described and the new species (*X. pradense* sp. nov., *X. paraiberica* sp. nov. and *X. iberica* sp. nov.) comprise a new complex of cryptic species (i.e. *X. macrodora* species complex) in which morphologically and morphometrically indistinguishable taxa appear within this genus of nematodes.

### Species separation based on ribosomal and mitochondrial DNA

Species separation using molecular markers demonstrated that *X. iberica* sp. nov., *X. paraiberica* sp. nov., and *X. pradense* sp. nov., were clearly separated among them and from *X. macrodora* from the USA and Italy. The ratio between intra- and inter-species molecular variation for D2-D3 expansion segments of 28S rRNA and ITS region of all three Spanish species was very low (0.01–0.05), whereas COI was higher in *X. macrodora* (0.25), *X. iberica* sp. nov. (0.12), and *X. iberica* sp. nov. (0.11) (Table 2), confirming that COI is highly diversified in USA populations. However, for all three new species, the D2D3 and ITS genes clearly showed intra- and inter-species molecular variation (Table 2), suggesting that the probability of species separation with these loci was high [37]. Likewise, the P ID (Liberal) values for all four species and loci were ≥ 0.95, suggesting that species can be adequately separated [37, 38]. The P ID (Liberal) value [37] represents the probability that a correct species identification would be made using best sequence alignment (BLAST), closest genetic distance, or placement on a tree (falling within or being sister to a monophyletic species clade). Species with P ID (Liberal) ≥ 0.93 were considered to be adequately delimited [38]. Additionally, all clade supports for the three loci were well-supported (PP = 1.00), except for *X. iberica* sp. nov. in COI marker (PP = 0.89) and Rosenberg’s PAB values also supported the monophyly (*P* < 0.05) of each of the four species separately [39].

**Table 2:** Parameters evaluating *Xenocriconemella* species complex delimitation based on two rRNA genes (D2-D3 expansion segments of the 28S rRNA, ITS) and one mtDNA barcoding locus, COI, for four *Xenocriconemella* species of the complex

**Species**	**Gene**	**Intra/Inter**^**a**^	**P ID (Liberal)**^**b**^	**Clade Support**^**c**^	**Rosenberg’s P**_**AB**_^**d**^
*Xenocriconemella macrodora*	D2-D3	-	-	-	-
	ITS	-	-	-	-
	COI	0.25	**0.97 (0.94, 1.0)** ^e^	**1.00**	**5.9 × 10**^−30^
*Xenocriconemella iberica* sp. nov.	D2-D3	0.05	**1.00 (0.97, 1.0)**	**1.00**	**4.4 × 10**^−17^
	ITS	0.01	**0.98 (0.88, 1.0)**	**1.00**	**1.9 × 10**^−4^
	COI	0.12	**0.99 (0.96, 1.0)**	**0.89**	**1.2 × 10**^−44^
*Xenocriconemella paraiberica* sp. nov.	D2-D3	0.03	**1.00 (0.97, 1.0)**	**1.00**	**3.9 × 10**^−35^
	ITS	0.02	**1.00 (0.96, 1.0)**	**1.00**	**3.2 × 10**^−8^
	COI	0.11	**0.99 (0.96, 1.0)**	**1.00**	**5.9 × 10**^−30^
*Xenocriconemella pradense* sp. nov.	D2-D3	0.03	**1.00 (0.97, 1.0)**	**1.00**	**0.01**
	ITS	0.01	**0.98 (0.88, 1.0)**	**1.00**	**1.9 × 10**^−4^
	COI	0.04	**1.00 (0.94, 1.0)**	**1.00**	**3.9 × 10**^−16^

^a^Intra-species variation relative to inter-species variation

^b^The P ID (Liberal) value represents the probability (with the 95% confidence interval) for the prediction of making a correct identification of an unknown specimen of the focal species using DNA Barcoding (closest genetic distance). P ID (Liberal) values ≥ 0.93 were considered to be delimited [38]. Numbers in bold represent significant values

^c^Clade support: posterior probabilities from Bayesian trees

^d^Rosenberg’s P_AB_ value is the probability that the monophyly of a group of sequences is the result of random branching

^e^Significant results are indicated in bold. (-) Not obtained or not performed because only a single sequence of D2-D3 or ITS for this species is available in NCBI

### Ribosomal and mitochondrial diversity within the *Xenocriconemella* species complex

Amplification of the D2-D3 expansion segments of 28S rRNA, 18S rRNA, ITS rRNA, and partial COI regions from the three new *Xenocriconemella* species and *Criconemoides rosmarini* yielded single fragments of approximately 900, 1000, and 400 bp, respectively, based on gel electrophoresis. Forty-five, 51 and 16 sequences from the D2-D3 region of the 28S rRNA were generated from 13 populations of *X. iberica* sp. nov. (OR880107-OR880151), 12 populations of *X. paraiberica* sp. nov. (OR880152-OR880202) and from three populations of *X. pradense* sp. nov. (OR880203-OR880218), respectively, showing very low intraspecific variation for this region. Only three variable positions for *X. iberica* sp. nov., two variable positions for *X. paraiberica* sp. nov., and no variable positions were detected in *X. pradense* sp. nov.

D2-D3 expansion segments of 28S rRNA sequences from the three new *Xenocriconemella* species are related but clearly dissimilar, with the unique accession available in GenBank for this genus, *X. macrodora* (AY780960, Italy) showing similarity values of 95% (differing by 28 nucleotides and four indels) with *X. pradense* sp. nov., 94% with *X. iberica* sp. nov. (32 nucleotides and four indels) and 90% with *X. paraiberica* sp. nov. (53 nucleotides and four indels). The species closest to *X. iberica* sp. nov. (OR880107-OR880151) was *X. pradense* sp. nov. (OR880203-OR880218), being 94% similar for the D2-D3 region (differing by 43–46 nucleotides and no indels). In the case of *X. paraiberica* sp. nov. (OR880152-OR880202), the closest species was *X. iberica* sp. nov., being 92% similar among them (differing by 56–58 nucleotides and no indels). Finally, *X. pradense* sp. nov. (OR880203-OR880218) showed the highest similarity values when compared with *X. macrodora* from Italy (AY780960), 95% similarity (differing by 28 nucleotides and four indels).

Intraspecific variation in the ITS rRNA gene detected among the three studied populations of *X. paraiberica* sp. nov. (OR878338-OR878349) varied from none to three nucleotides (99% similarity and no indels). However, no intraspecific variation for this region was detected between the studied populations of *X. iberica* sp. nov (OR878332-OR878337) and *X. pradense* sp. nov. (OR878350-OR878355) ITS rRNA aligned sequences of *Xenocriconemella* species from the Iberian Peninsula were dissimilar in a wide range from 139 to 204 nucleotides (15–18%). This range increases when including the sequence of *X. macrodora* from the USA (JQ708139), up to 25% (101–115 nucleotides and 40–45 indels).

The 18S rRNA from the three new *Xenocriconemella* spp. (OR878356- OR878361) described here showed high similarity values (98.4–99% similarity, from 10 to 30 nucleotides and four indels) among them, and with all accessions from *X. macrodora* deposited in GenBank, including one accession most probably misidentified as *X. macrodora* from Portugal (MT229843, , differing by 8–11 bp). Unfortunately, no more molecular data were available in GenBank from this population and further studies will be needed for clarifying this identification. No intraspecific variability was detected for this marker in any populations included in the present study.

**Fig. 3:**
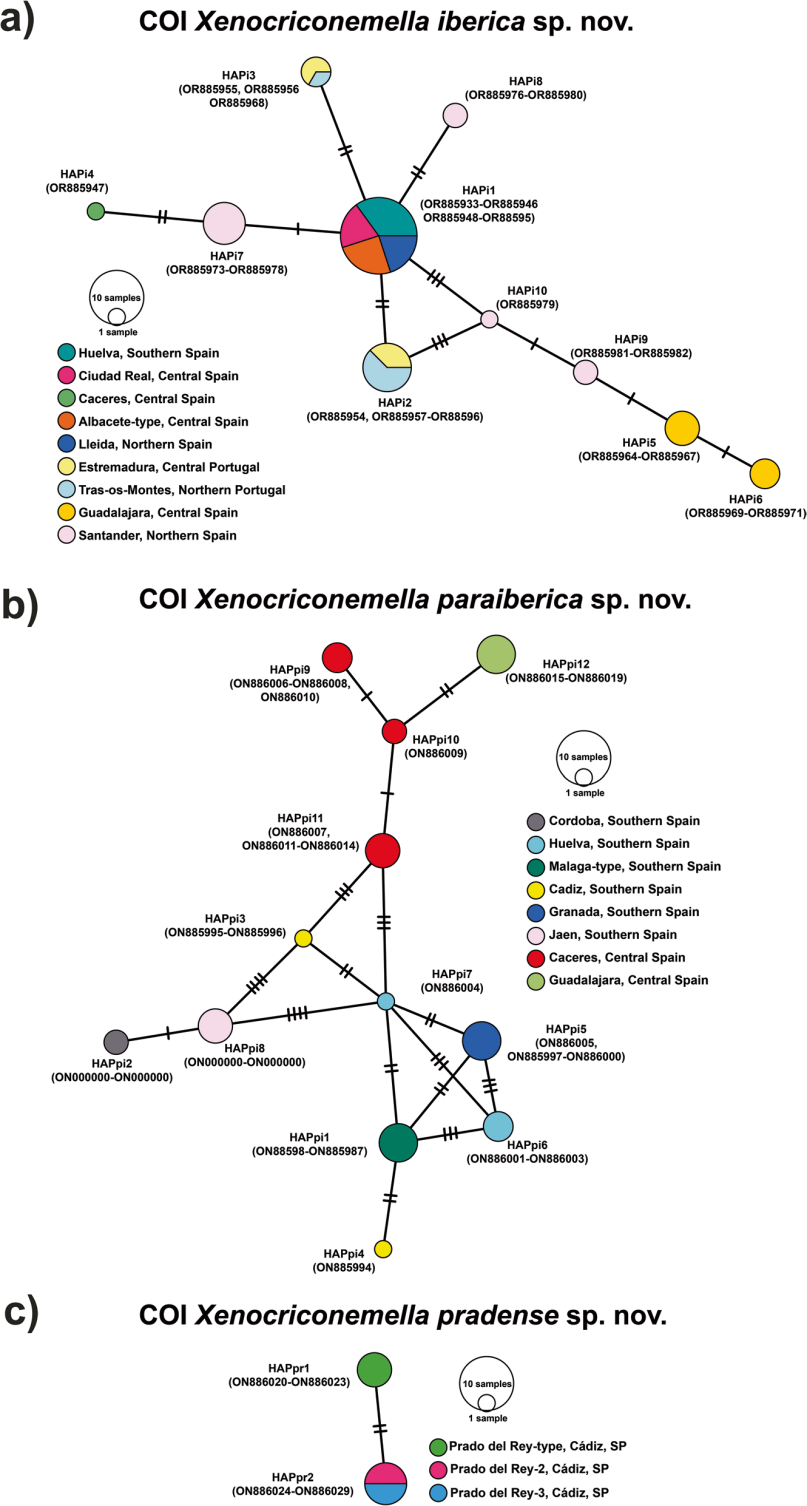
TCS network analysis of partial mitochondrial COI haplotypes of the *Xenocriconemella macrodora* species complex. **a** *Xenocriconemella iberica* sp. nov. COI haplotypes; (**b**) *Xenocriconemella paraiberica* sp. nov. COI haplotypes; (**c**) *Xenocriconemella pradense* sp. nov. COI haplotypes. Coloured circles embody haplotypes for each geographic sampling region, and their diameter is proportionate to the number of individuals sharing the same haplotype. Black short lines on the branches specify the numbers of mutated nucleotides in the alignment that separate each haplotype

Ninety-seven new COI sequences were obtained in this study, 50 from *X. iberica* sp. nov. (OR885933-OR885982), 37 from *X. paraiberica* sp. nov. (OR885983-OR886019), and 10 from *X. pradense* sp. nov. (OR886020-OR886029). These partial COI sequences were clearly different from the COI sequences of *X. macrodora* from the USA deposited in GenBank [5], being from 88 to 92% similar (from 26 to 32 nucleotides in difference), including the accessions from specimens collected near the type locality MN711389, MF094906–MF094907 (Long Branch Stream Valley Park, Accotink Watershed, Fairfax County, Virginia). Intraspecific variation for the partial COI region within the Iberian *Xenocriconemella* species was higher than that for the D2-D3 region but, in any case, not more than 1%. fifty aligned sequences from 13 populations of *X. iberica* sp. nov. (OR885933-OR885982) showed 13 variable positions, resulting in 10 different haplotypes (Fig. 3). HAPi1 was the most common and prevalent, grouping 20 sequences from four populations located in four different provinces at northern, central and southern Spain, including Lleida (PIR17), Albacete (COT22), Ciudad Real (FCQ02) and Huelva (HUC01). The 11 sequences from Portugal generated 2 haplotypes, HAPi2 and HAPi3 with eight and three sequences, respectively, both of which were detected in the northern and central regions (provinces of Tras-os-Montes and Estremadura, respectively). HAPi4 corresponds to the unique sequences from *X. iberica* sp. nov. detected in Cáceres Province (GUR05). HAPi5 and HAPi6 grouped seven sequences from the two populations from Guadalajara Province (XN48B and XN56B). Finally, populations from Santander Province (HCANT and RCANT) were the most variable, with four haplotypes, HAPi7 and HAPi8 with six and two sequences from both populations, and HAPi9 and HAPi10 with two and one sequences from the Gismana (RCANT) population. Intraspecific variability observed in the 37 sequences from *X. paraiberica* sp. nov. (OR885983-OR886019) was similar to *X. iberica* sp. nov., 15 variable positions were detected in the partial COI sequences included in this study. These variations were grouped into 12 different haplotypes (Fig. 3). However, any haplotype was predominant in the sampled populations and each haplotype had its own specific province (Fig. 3). HAPpi1 corresponds to the sequences from the type locality, Casares, Málaga province, (CAS22), and HAPpi2 includes the two sequences belonging to the Córdoba population (COA01). HAPpi3 and HAPpi4 grouped three sequences from one population from Cádiz Province (CAC01). The five sequences from the two Granada populations (BUQ1 and CNR01) comprise the haplotype HAPpi5. Sequences from two populations from Huelva Province (HUE00 and HUA03) gave rise in two different haplotypes, HAPpi6 and HAPpi7, formed by 3 and 1 sequences, respectively. Populations from Jaén (CZQ05) and Guadalajara (XN43B and XN55A) provinces yield only one haplotype each, HAPpi8 and HAPpi12, respectively. Last, sequences from Caceres Province (GUR03 and GUR04) were grouped into three different haplotypes, HAPpi9, three sequences from sample GUR04, HAPpi10 two sequences from sample GUR04, and HAPpi11, four sequences from sample GUR03.

Finally, the ten sequences from *X. pradense* sp. nov. (OR886020-OR886029) showed an intraspecific variability of only two nucleotides, which grouped into two haplotypes, one of them (HAPpr1) with the six sequences from the type population (GRQ01) and the other (HAPr2) with the four sequences from populations GRQ02 and GRQ05.

Molecular data from *Criconemoides rosmarini* were obtained for the first time in the present study. Specifically, two new sequences from the D2-D3 expansion segments of 28S rRNA (OR880219-OR880220) and one sequence from the partial COI region (OR886030) were identified. The closest species to *C. rosmarini* was *Discocriconemella limitanea* (MZ262311-MZ262314, MT159832), being 90% similar and varying from 67 to 69 nucleotides and one indel for the D2-D3 expansion segments of 28S rRNA and 85% similar, from 104 to 101 nucleotides and 8 indels, for the partial COI. D2-D3 expansion segments of 28S rRNA and COI sequences from *C. rosmarini* differed significantly from those of the *Xenocriconemella* species complex, showing similarity values of 83% for the D2-D3 expansion segments of 28S rRNA (117-119 nucleotides and no indels), and from 79 to 81% (57-60 nucleotides and five indels) for the partial COI.

### Phylogeny

**Fig. 4:**
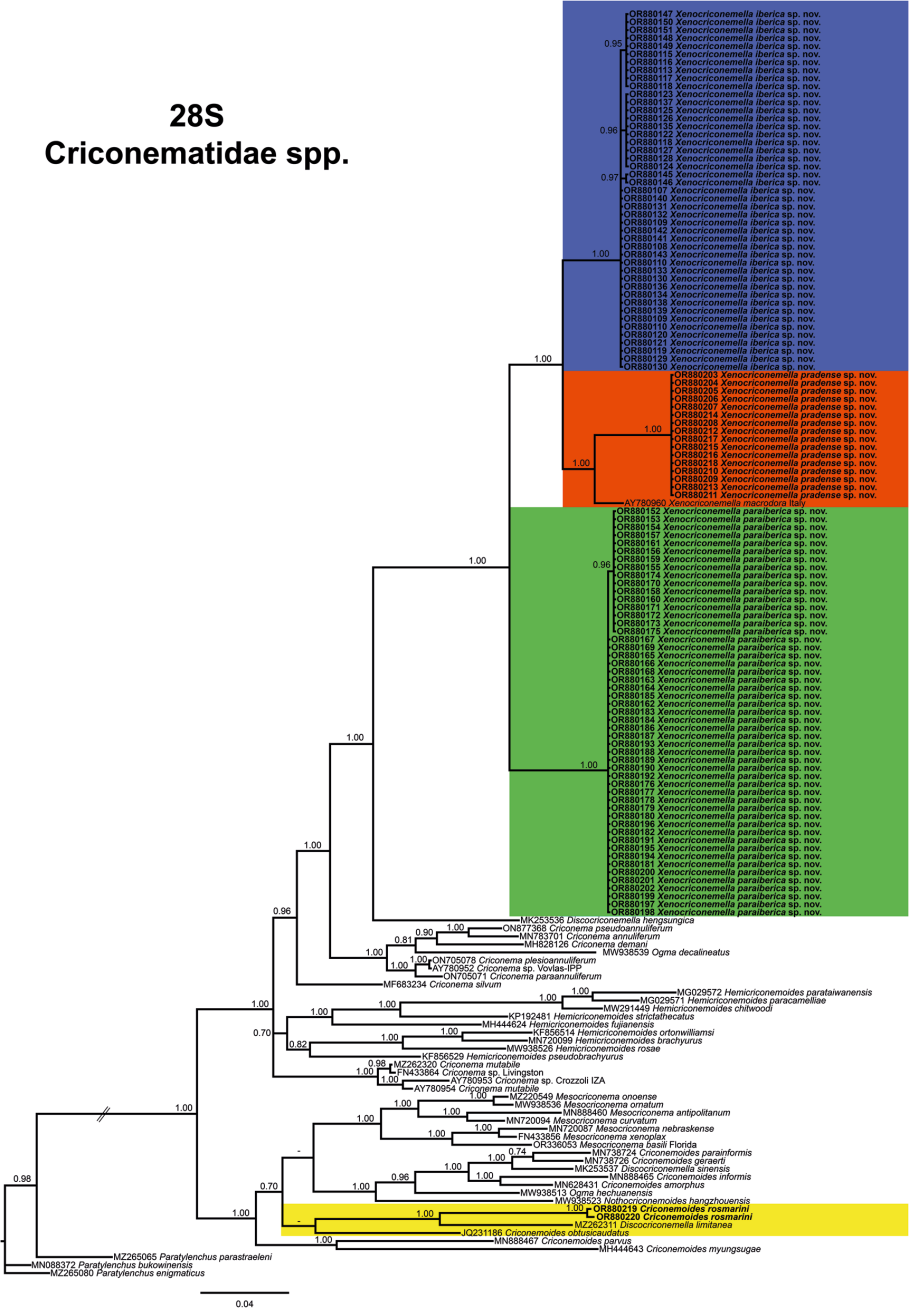
Phylogenetic relationships within the genus *Xenocriconemella*. Bayesian 50% majority rule consensus tree as inferred from D2-D3 expansion domains of the 28S rRNA sequence alignment under the general time-reversible model with invariable sites and a gamma-shaped distribution (GTR + I + G). Posterior probabilities of more than 0.70 are given for appropriate clades. Newly obtained sequences in this study are shown in bold. The scale bar indicates expected changes per site, and the coloured boxes indicate the clade association of the *Xenocriconemella macrodora* species complex

A total of 158 sequences from the D2-D3 domains of the 28S rRNA gene alignment (697 bp long) were included. Three outgroup species (*Paratylenchus bukowinensis* (MN088372), *Paratylenchus enigmaticus* (MZ265080), and *Paratylenchus parastraeleni* (MZ265065)) were included in the analysis. The Bayesian 50% majority rule consensus tree inferred from the D2-D3 alignment is given in Fig. 4. For this region, all species that belong to the *X. macrodora* species complex clustered together in a well-supported (PP = 1.00) clade, which was subdivided into two subclades, one of them (PP = 1.00) formed by *X. paraiberica* sp. nov. (OR880152-OR880202) and the other one (PP = 1.00) by *X. iberica* sp. nov (OR880107-OR880151), *X. pradense* sp. nov (OR880203-OR880218) and *X. macrodora* from Italy (AY780960). In this analysis, *X. pradense* sp. nov (OR880203-OR880218) and *X. macrodora* (AY780960) clustered together, although clearly separated, in a well-supported subclade (PP = 1.00). *Criconemoides rosmarini* (OR880219–OR880220) appears to occupy a basal position in a well-supported clade (PP = 1.00) with *Discocriconemella limitanea* (MZ262311) and these two species are included in a not well-supported subclade with *Criconemoides obtusicaudatus* (JQ231186).

**Fig. 5:**
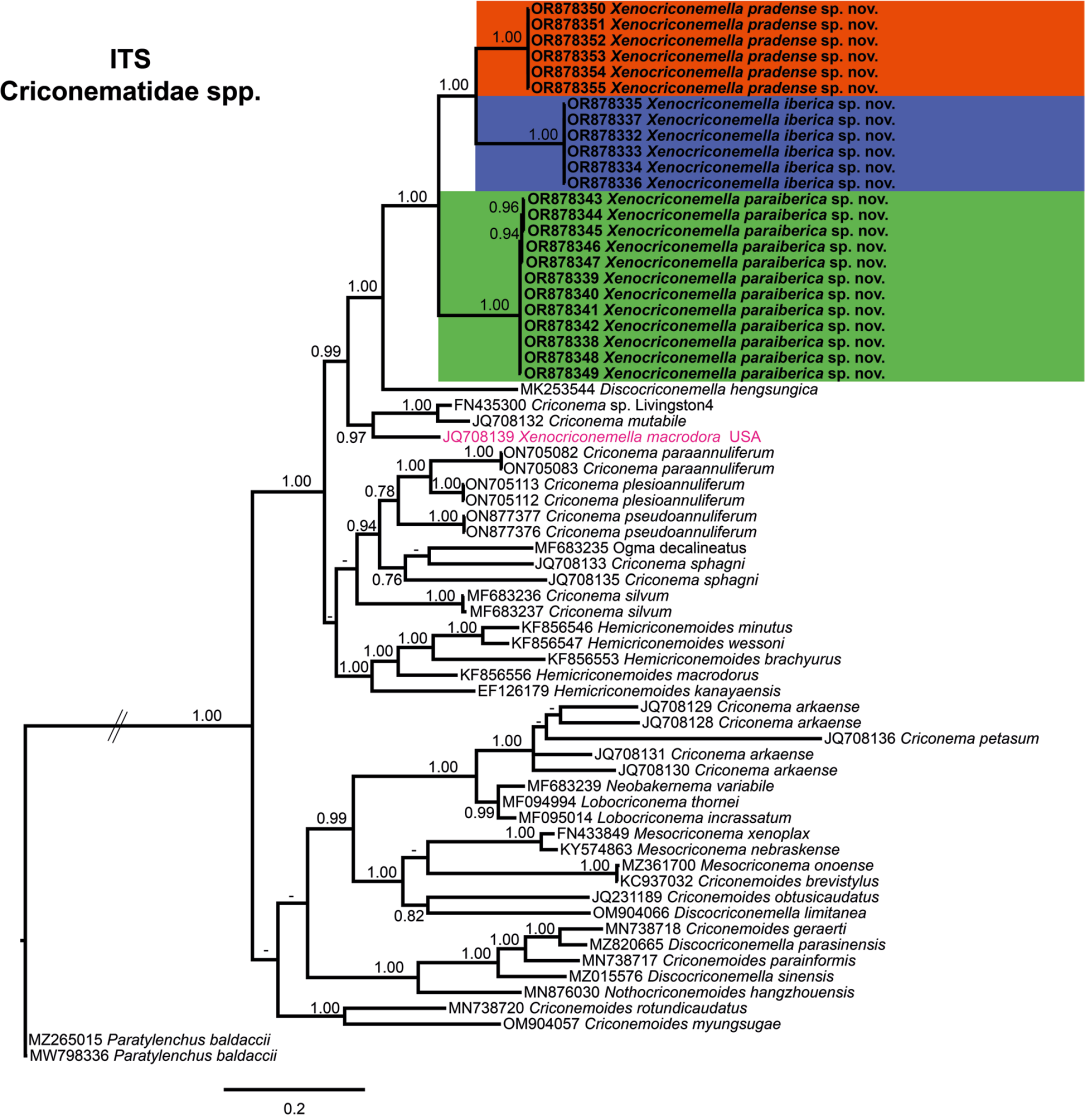
Phylogenetic relationships within the genus *Xenocriconemella*. Bayesian 50% majority rule consensus tree as inferred from ITS rRNA sequence alignment under the transversion model with invariable sites and a gamma-shaped distribution (TVM + I + G). Posterior probabilities of more than 0.70 are given for appropriate clades. Newly obtained sequences in this study are shown in bold. The scale bar indicates expected changes per site, and the coloured boxes indicate the clade association of the *Xenocriconemella macrodora* species complex

The phylogenetic position of *X. iberica* sp. nov., *X. paraiberica* sp. nov. and *X. pradense* sp. nov. in the ITS region tree is given in Fig. 5. The phylogenetic tree based on ITS Criconematidae spp. sequences resolved a well-supported major clade (PP = 1.00), including a subclade not well-supported with *Hemicriconemoides* spp., some *Criconema* spp., and *Ogma decalineatus* (MF683235), and a well-supported subclade (PP = 0.99) including *Xenocriconemella* spp. together with *Discocriconemella hengsungica* (MK253544), *Criconema mutabile* (JQ708132) and *Criconema* sp. Livingston4 (FN435300). *Xenocriconemella iberica* sp. nov. (OR878332-OR878337), *X. paraiberica* sp. nov. (OR878338-OR878349) and X*. pradense* sp. nov. (OR878350-OR878355) clustered together in a well-supported clade (PP = 1.00) and these species clustered with *Discocriconemella hengsungica* (MK253544) in a well-supported clade (PP=1.00), but were clearly separated from the unique ITS of *X. macrodora* from the USA (JQ708139).

**Fig. 6:**
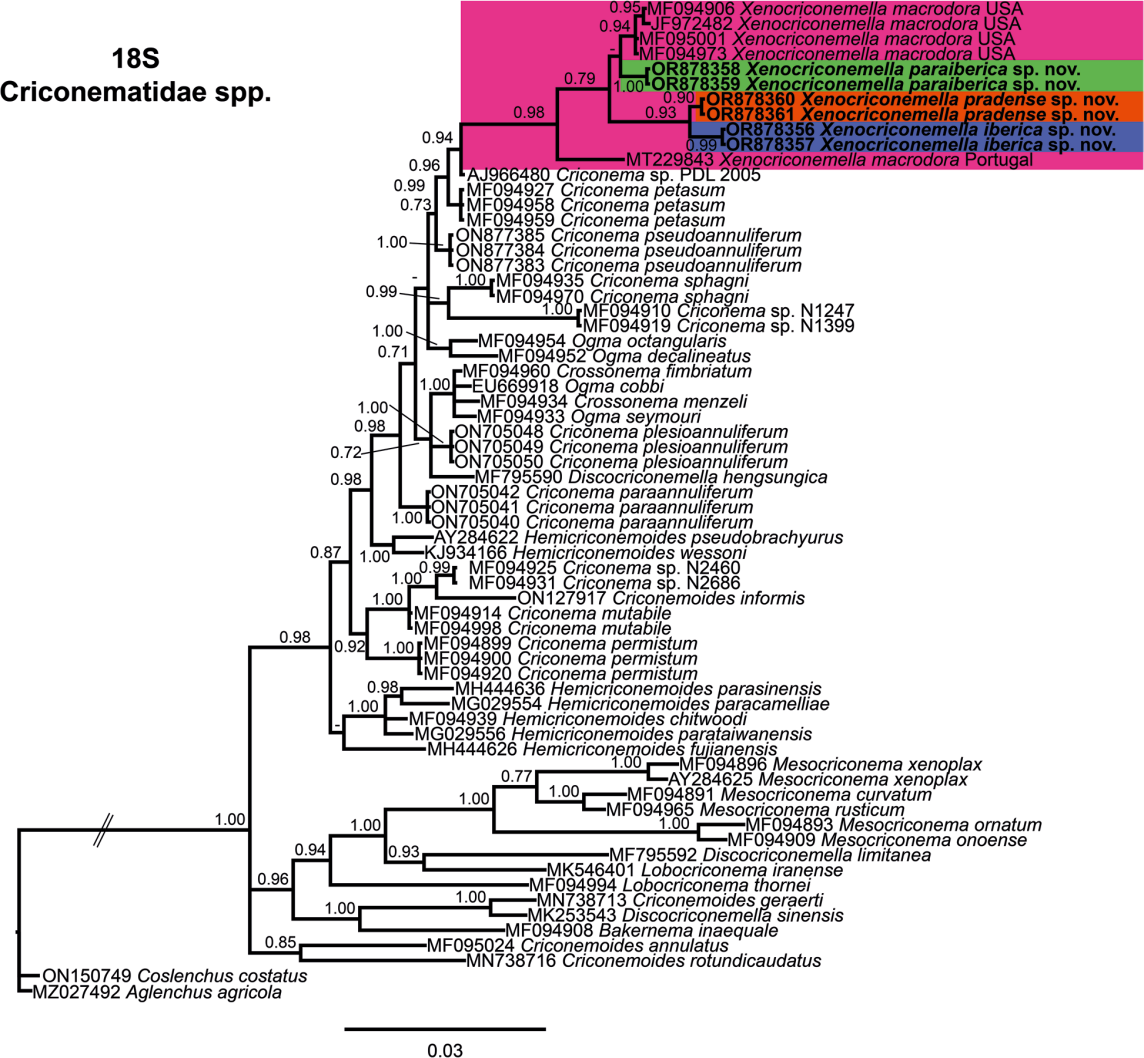
Phylogenetic relationships within the genus *Xenocriconemella*. Bayesian 50% majority rule consensus tree as inferred from 18S rRNA sequence alignment under the transition model with invariable sites and a gamma-shaped distribution (TIM2ef + I + G). Posterior probabilities of more than 0.70 are given for appropriate clades. Newly obtained sequences in this study are shown in bold. The scale bar indicates expected changes per site, and the coloured boxes indicate the clade association of the *Xenocriconemella macrodora* species complex

The 18S rRNA gene alignment (1660 bp long) included six new sequences: two sequences from *X. iberica* sp. nov. (OR878356-OR878357), two sequences from *X. paraiberica* sp. nov. (OR878358-OR878359), and two sequences from *X. pradense* sp. nov. (OR878360-OR878361) see Fig. 6. Accessions from the three new species and *X. macrodora* from the USA and Portugal clustered together in a moderately-supported clade (PP= 0.98). However, the different branches in which these subclades are not well-supported.

**Fig. 7:**
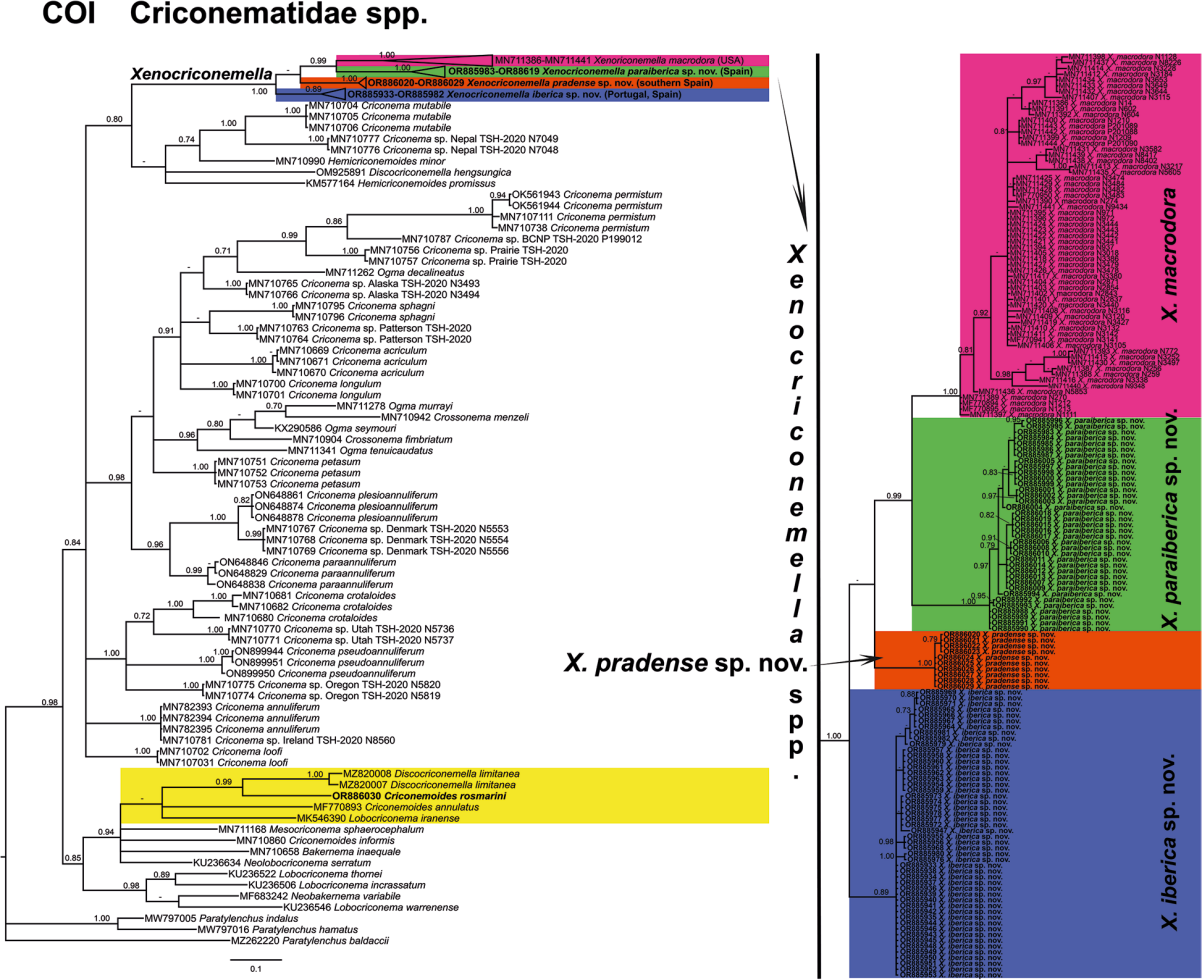
Phylogenetic relationships within the genus *Xenocriconemella*. Bayesian 50% majority rule consensus tree as inferred from cytochrome c oxidase subunit 1 (COI) sequence alignment under the transition model with invariable sites and a gamma-shaped distribution (TIM3 + I + G). Posterior probabilities of more than 0.70 are given for appropriate clades. Newly obtained sequences in this study are shown in bold. The scale bar indicates expected changes per site, and the coloured boxes indicate the clade association of the *Xenocriconemella macrodora* species complex

The COI gene alignment (323 bp long) included 106 new sequences: 50 sequences from *X. iberica* sp. nov. (OR885933-OR885982), 37 sequences from *X. paraiberica* sp. nov. (OR885983-OR886019), 10 sequences from *X. pradense* sp. nov. (OR886020-OR886029) and one sequence from *C. rosmarini* (OR886030). Finally, *Paratylenchus baldaccii* (MZ262220), *Paratylenchus hamatus* (MW797016) and *Paratylenchus indalus* (MW797005) were used as outgroups. The Bayesian 50% majority rule consensus tree inferred from the COI sequence alignment is given in Fig. 7. For COI, *Xenocriconemella* spp. clustered together in a well-supported clade (PP= 1.00), with the difference that for this marker, *X. iberica* sp. nov. is the only species that appears alone in a weakly supported subclade (PP= 0.89) while *X. pradense* and *X. macrodora* from the USA clustered together in a well-supported subclade (PP = 0.99), but the phylogenetic relationship of these species with *X. pradense* sp. nov. is not well-defined and remains unresolved. However, each species is well-separated in its own clade (PP = 1.00). Once again, *C. rosmarini* (OR886030) appears in a well-supported clade (PP = 0.99) with *Discocriconemella limitanea* (MZ820007-MZ820008)*.*

## Taxonomic account

### *Xenocriconemella iberica* sp. nov. (Figs. 8, 9, 10 and 11; Tables 3, 4)

#### Zoobank

**Fig. 8:**
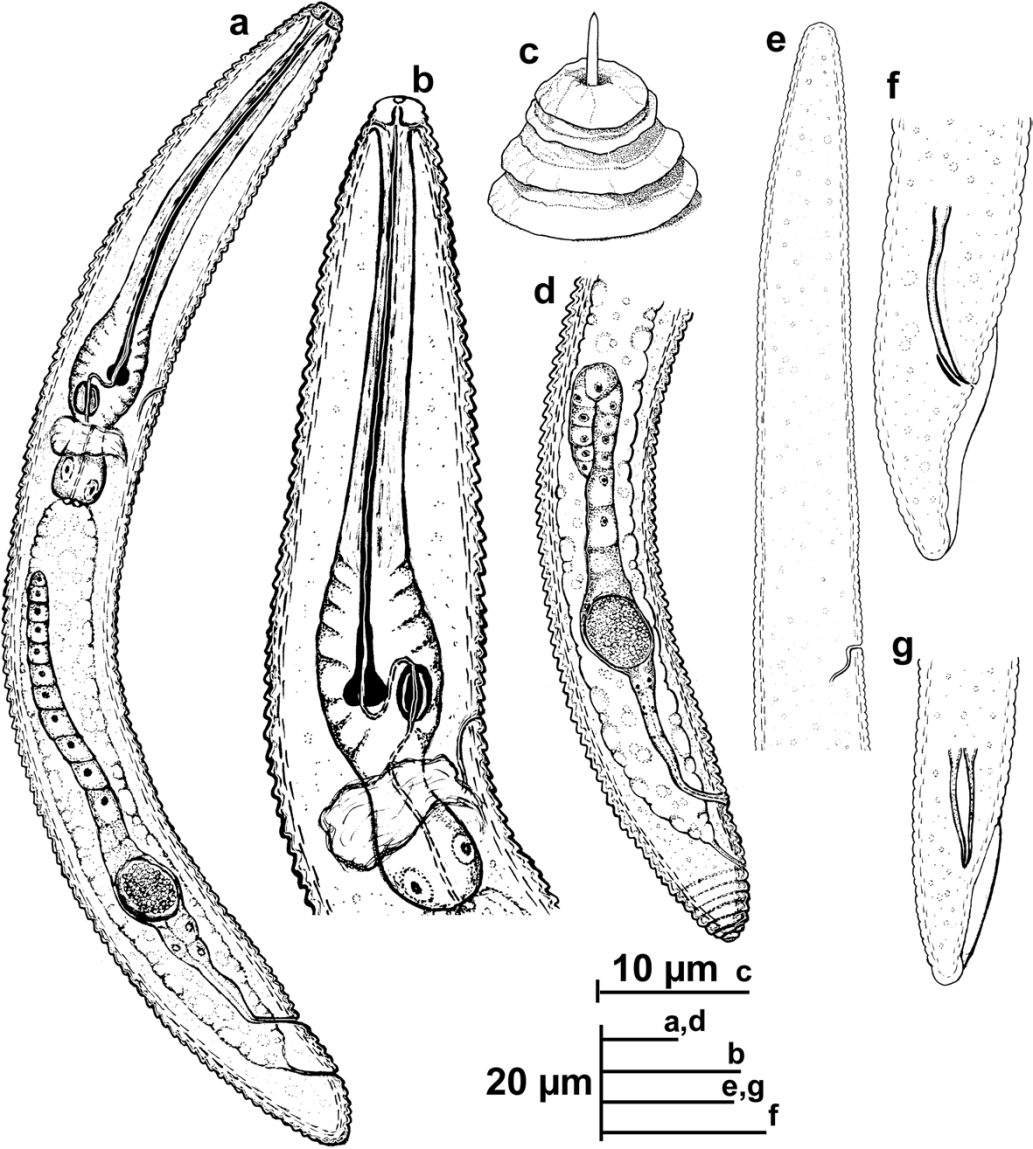
Line drawings of *Xenocriconemella iberica* sp. nov. **a** whole female, (**b**) female anterior region; (**c**) detail of *en face* view; (**d**) female posterior region; (**e**) male anterior region showing absence of stylet; (**f**, **g**) male posterior region

**Fig. 9:**
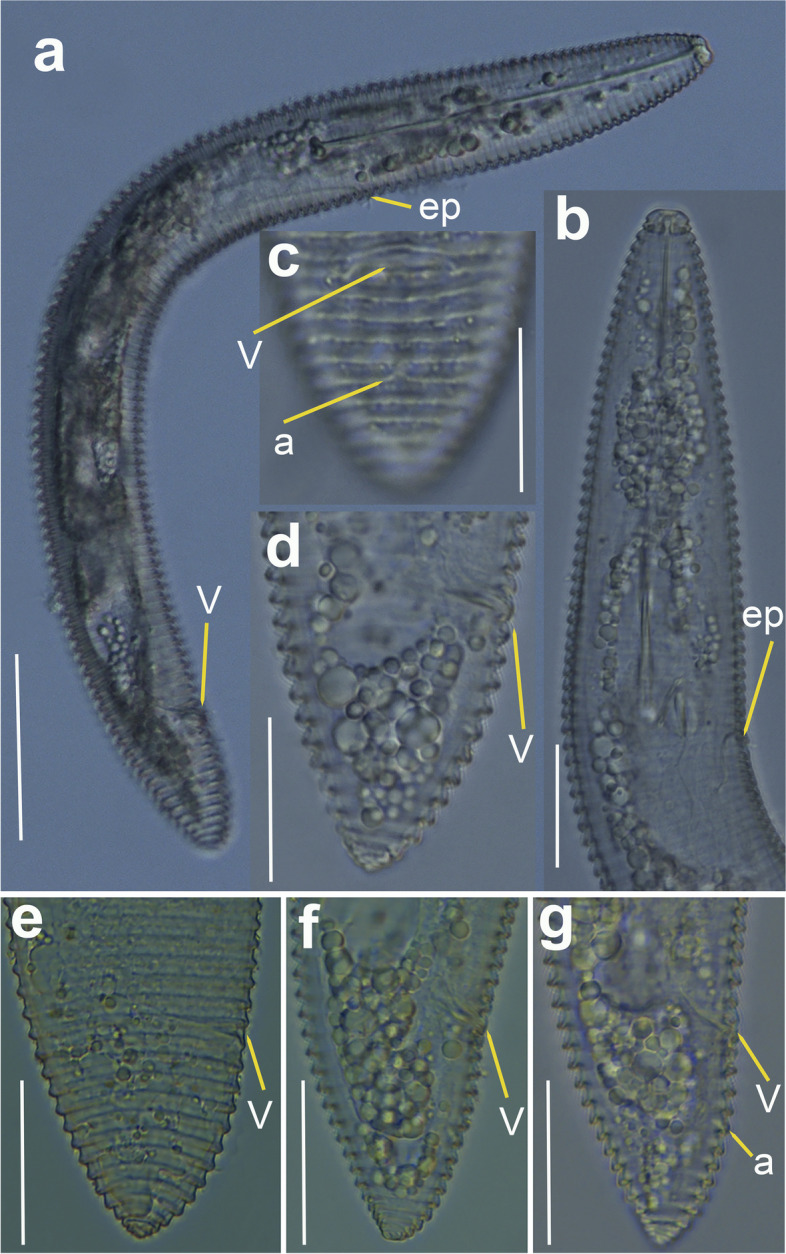
Light micrographs of *Xenocriconemella iberica* sp. nov. females. **a** whole body; (**b**) pharyngeal region showing excretory pore (arrow); (**c**-**g**) posterior region showing vulva and anus (arrow). Abbreviations: a = anus; ep = excretory pore; V = vulva. Scale bars: (**a**) = 50 µm; (**b**-**g**) = 20 µm

**Fig. 10:**
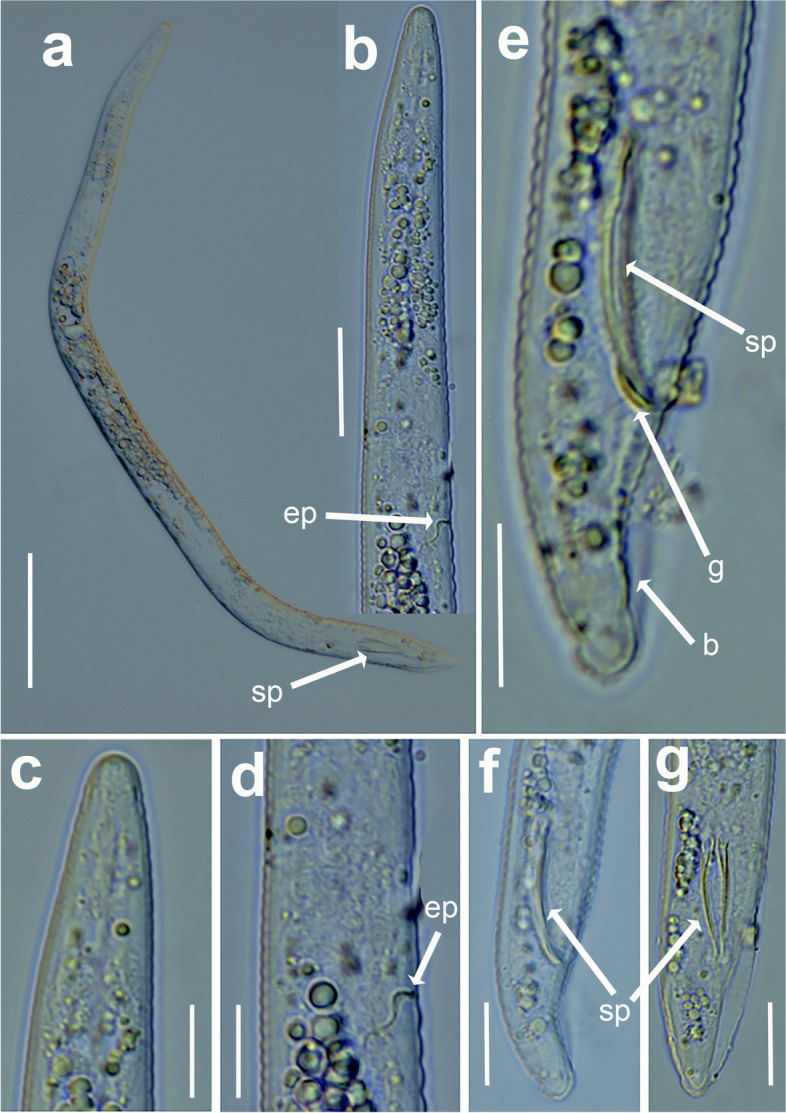
Light micrographs of *Xenocriconemella iberica* sp. nov. male. **a**, whole body, showing spicules (arrow); (**b**, **c**), anterior region showing absence of stylet and undifferentiated pharynx; (**d**), detail of excretory pore (arrow); (**e**-**g**), male tail showing spicules, gubernaculum and bursa (arrow). Abbreviations: b = bursa; ep = excretory pore; g = gubernaculum; sp = spicules. Scale bars: (**a**) = 50 µm; (**b**) = 20 µm; (**c**–**g**) = 10 µm

**Fig. 11:**
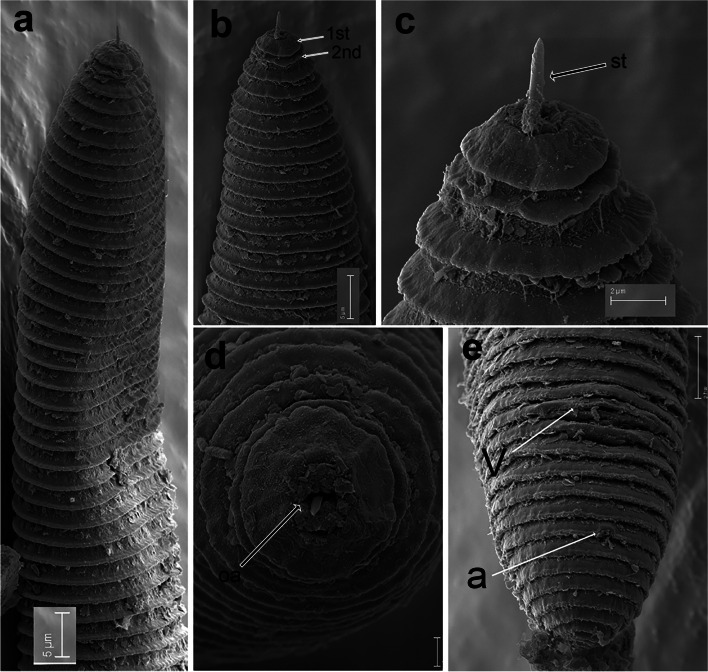
SEM micrographs of *Xenocriconemella iberica* sp. nov. female. **a** anterior region; (**b**, **c**) lip region showing 1st and 2nd body annuli; (**d**) en face view showing oral aperture (arrow); (**e**) posterior region in frontal view showing vulva and anus (arrow). Abbreviations: a = anus; oa = oral aperture; V = vulva; 1st, 2nd = first- and second-body annuli. Scale bars: (**a**, **b**) = 5 µm; (**c**) = 2 µm; (**d**) = 1 µm; (**e**) = 5 µm

urn:lsid:zoobank.org:act:18F75622-A236-4448-AC13-EA2E3777072A Figs. 8, 9, 10 and 11; Tables 3, 4.

**Table 3:** Morphometrics of *Xenocriconemella iberica* sp. nov. from the rhizosphere of forest trees in several localities of the Iberian Peninsula

		**Paratype**						
**Character/Ratio**^**a**^	**Holotype**	**Females**	**Females**	Males*	**Females**	**Females**	**Females**	**Females**
Sample code^b^	COT22	COT22	HUC01	HUC01	PIR17	FCQ02	GUR05	PTA01
n	1	20	5	2	5	5	2	5
L	289	279.0 ± 20.3 (251-327)	302.8 ± 12.9 (284-316)	(312, 370)	282.0 ± 26.1 (254-322)	298.4 ± 24.1 (266-328)	(280, 290)	313.4 ± 38.2 (258-350)
R	106	107.6 ± 2.1 (103-111)	104.2 ± 1.1 (103-105)	-	107.2 ± 3.0 (104-112)	112.2 ± 5.4 (106-118)	(108, 109)	104.2 ± 3.7 (99-108)
Rst	35	35.3 ± 1.0 (34-37)	37.2 ± 1.6 (35-39)	-	38.4 ± 2.7 (35-42)	39.2 ± 4.0 (34-43)	(34, 34)	37.4 ± 2.1 (35-40)
Roes	45	43.1 ± 2.0 (40-47)	46.8 ± 2.4 (43-49)	-	48.2 ± 3.1 (45-52)	49.2 ± 2.6 (46-52)	(42, 42)	47.4 ± 2.1 (45-50)
Rex	36	36.4 ± 0.7 (35-37)	38.2 ± 1.6 (36-40.4	-	40.2 ± 3.3 (36-44)	39.2 ± 2.6 (36-42)	(35, 36)	38.4 ± 2.1 (36-41)
RV	13	12.7 ± 0.7 (11-14)	15.4 ± 0.9 (14-16)	-	13.4 ± 1.1 (12-15)	14.4 ± 1.8 (12-16)	(13, 13)	14.4 ± 1.5 (12-16)
Rvan	4	4.1 ± 0.3 (4-5)	4.0 ± 0.0 (4)	-	4.8 ± 0.4 (4-5)	4.0 ± 0.0 (4)	(4, 4)	4.0 ± 0.0 (4)
Ran	9	8.6 ± 0.7 (7-9)	11.4 ± 0.9 (10-12)	-	8.6 ± 0.9 (8-10)	10.4 ± 1.8 (8-12)	(9, 9)	10.4 ± 1.5 (8-12)
O	10.0	8.6 ± 1.3 (6.8-11.6)	8.1 ± 0.6 (7.3-8.7)	-	8.9 ± 1.3 (6.8-10.0)	8.1 ± 0.8 (6.9-9.1)	(8.0, 8.8)	8.0 ± 0.2 (7.8-8.2)
a	9.6	10.5 ± 0.7 (8.8-11.7)	9.5 ± 0.6 (7.3-8.7)	(18.4, 21.8)	10.5 ± 0.7 (9.6-11.5)	10.3 ± 0.7 (9.1-11.0)	(10.4, 10.4)	9.3 ± 1.3 (7.8-11.2)
b	2.4	2.4 ± 0.1 (2.2-2.7)	2.3 ± 0.1 (2.2-2.5)	(2.7, 4.1)	2.4 ± 0.1 (2.2-2.4)	2.4 ± 0.1 (2.2-2.5)	(2.4, 2.3)	2.2 ± 0.2 (2.0-2.5)
c	19.3	20.0 ± 3.1 (15.7-27.3)	15.3 ± 2.4 (12.1-18.4)	(14.2, 14.8)	20.3 ± 2.2 (17.5-23.0)	18.9 ± 2.2 (16.6-21.9)	(20.0, 22.3)	16.1 ± 0.9 (15.2-17.5)
c’	0.8	0.8 ± 0.06 (0.7-0.9)	1.0 ± 0.08 (0.9-1.1)	(2.0, 1.8)	0.8 ± 0.05 (0.8-0.9)	0.9 ± 0.05 (0.8-0.9)	(0.8, 0.8)	0.9 ± 0.05 (0.8-1.0)
V or T	89.6	89.7 ± 1.0 (88.6-91.9)	88.4 ± 0.9 (87.5-89.6)	(38.8, 68.1)	90.1 ± 1.1 (89.1-91.7)	89.4 ± 1.2 (88.3-91.4)	(91.1, 91.7)	88.8 ± 0.9 (88.1-90.3)
VL/VB	1.3	1.2 ± 0.1 (0.7-1.4)	1.3 ± 0.1 (1.1-1.4)	-	1.2 ± 0.3 (0.7-1.4)	1.1 ± 0.3 (0.7-1.4)	(1.2, 1.2)	1.2 ± 0.1 (1.1-1.4)
Stylet	90.0	90.4 ± 3.6 (86.5-97.5)	93.4 ± 2.6 (90.0-96.0)	-	89.9 ± 2.2 (87.0-92.0)	96.8 ± 5.3 (88.0-101.0)	(88.0, 91.0)	95.2 ± 5.8 (89.0-103.0)
Conus	84.0	84.2 ± 3.4 (80.0-90.0)	86.0 ± 2.7 (82.0-89.0)	-	83.6 ± 2.3 (80.0-86.0)	88.8 ± 5.1 (80.0-93.0)	(81.0, 83.0)	87.6 ± 5.0 (82.0-94.0)
Pharynx	122.0	116.5 ± 11.0 (102-143)	131.4 ± 3.8 (128-138)	(114, 90)	119.6 ± 10.7 (108-132)	126.0 ± 11.9 (106-138)	(116, 124)	140.8 ± 7.3 (132-150)
Max. body width	30.0	26.6 ± 1.9 (23.0-30.0)	31.9 ± 1.0 (30.5-33.0)	(17.0, 17.0)	26.8 ± 2.4 (24.0-30.0)	29.2 ± 4.2 (25.0-36.0)	(27.0, 28.0)	34.2 ± 5.4 (26.0-41.0)
Anal body diam.	18.0	17.6 ± 1.4 (15.5-21.0)	19.5 ± 1.9 (18.0-22.5)	(11.0, 14.0)	17.2 ± 1.6 (15.0-19.0)	18.6 ± 2.5 (16.0-21.0)	(17.0, 17.0)	21.6 ± 2.9 (16.0-23.0)
Vulva to anus distance	18.0	13.4 ± 3.6 (8.0-24.0)	13.6 ± 1.8 (11.0-16.0)	-	14.3 ± 2.4 (11.4-18.0)	14.7 ± 2.3 (11.5-17.0)	(16.0, 15.0)	15.4 ± 1.8 (13.0-17.0)
Tail	15.0	14.2 ± 1.8 (11.0-17.0)	20.1 ± 2.8 (17.0-24.5)	(22.0, 25.0)	14.0 ± 1.6 (12.0-16.0)	16.0 ± 2.4 (13.0-19.0)	(14.0, 13.0)	19.6 ± 2.9 (16.0-23.0)
Spicules	-	-	-	(22.0, 21.0)	-	-	-	-
Gubernaculum	-	-	-	(6.0, 5.0)	-	-	-	-

^a^Measurements are in µm and in the form: (mean) ± (standard deviation), (range). (-) Not obtained or not performed

^b^See Table 1 for identifying the sampled localities

^*^Identification confirmed by ribosomal (28S and ITS) and mitochondrial (COI) markers in both male specimens

**Table 4:** Morphometrics of *Xenocriconemella iberica* sp. nov. from the rhizosphere of forest trees in several localities of the Iberian Peninsula

**Character/Ratio**^**a**^	**Females**	**Females**	**Females**	**Females**	**Females**	**Females**	**Females**
Sample code^b^	PTQ01	PTA02	XEN37	XN48B	XN56B	HCANT	RCANT
n	5	2	5	5	5	5	5
L	296.0 ± 24.1 (271-325)	(284, 279)	311.6 ± 17.4 (282-325)	312.4 ± 4.9 (304-316)	277.4 ± 18.6 (246-294)	311.4 ± 14.1 (294-330)	301.4 ± 7.0 (289-306)
R	101.8 ± 4.4 (97-107)	(103, 100)	109.0 ± 4.5 (102-114)	110.0 ± 2.9 (105-112)	105.6 ± 4.8 (99-110)	112.4 ± 4.3 (108-119)	106.0 ± 1.6 (104-108)
Rst	37.6 ± 3.0 (35-42)	(40, 39)	34.6 ± 1.7 (33-37)	36.0 ± 0.7 (35-37)	35.2 ± 2.2 (32-37)	36.4 ± 2.7 (33-39)	37.8 ± 2.6 (35-41)
Roes	47.0 ± 3.9 (43-52)	(50, 49)	47.4 ± 2.3 (45-51)	48.8 ± 0.8 (48-50)	46.4 ± 3.2 (41-49)	48.4 ± 2.7 (45-51)	49.6 ± 1.5 (48-52)
Rex	38.6 ± 3.0 (36-43)	(41, 40)	35.6 ± 1.7 (34-38)	37.0 ± 0.7 (36-38)	35.8 ± 2.7 (32-38)	37.6 ± 2.4 (35-40)	38.8 ± 2.6 (36-42)
RV	14.6 ± 1.5 (12-16)	(16, 16)	13.2 ± 0.8 (12-14)	14.4 ± 0.5 (14-15)	13.2 ± 0.8 (12-14)	14.4 ± 0.5 (14-15)	13.8 ± 0.4 (13-14)
Rvan	3.8 ± 0.4 (3-4)	(4, 4)	4.0 ± 0.0 (4)	4.0 ± 0.0 (4)	4.0 ± 0.0 (4)	4.2 ± 0.4 (4-5)	4.0 ± 0.0 (4)
Ran	10.8 ± 1.6 (8-12)	(12, 12)	9.2 ± 0.8 (8-10)	10.4 ± 0.5 (10-11)	9.2 ± 0.8 (8-10)	10.2 ± 0.8 (9-11)	9.8 ± 0.4 (9-10)
O	7.6 ± 0.6 (7.1-8.5)	(7.8, 8.8)	7.7 ± 0.6 (7.2-8.8)	7.4 ± 0.4 (7.1-8.0)	8.3 ± 0.6 (7.6-9.1)	7.5 ± 0.5 (7.0-8.2)	7.4 ± 0.5 (7.0-8.2)
a	9.9 ± 1.4 (7.9-11.8)	(9.8, 10.0)	8.9 ± 1.9 (6.4-11.5)	9.9 ± 1.0 (8.2-10.9)	9.8 ± 0.4 (9.3-10.1)	9.7 ± 1.7 (8.2-11.9)	9.7 ± 1.3 (7.4-10.4)
b	2.2 ± 0.1 (2.0-2.3)	(2.4, 2.3)	2.3 ± 0.2 (2.0-2.5)	2.4 ± 0.1 (2.2-2.6)	2.3 ± 0.1 (2.2-2.4)	2.3 ± 0.2 (2.1-2.5)	2.2 ± 0.0 (2.2-2.3)
c	16.3 ± 1.6 (14.4-18.7)	(18.9, 17.4)	18.2 ± 2.8 (14.2-21.7)	16.7 ± 1.9 (14.3-19.0)	18.2 ± 0.7 (17.5-19.2)	18.6 ± 2.0 (15.5-20.6)	16.3 ± 1.2 (14.6-17.9)
c’	0.9 ± 0.07 (0.8-1.0)	(0.7, 0.8)	0.8 ± 0.11 (0.6-0.9)	0.8 ± 0.11 (0.7-0.9)	0.8 ± 0.06 (0.7-0.9)	0.8 ± 0.12 (0.7-1.0)	0.9 ± 0.04 (0.9-1.0)
V or T	88.4 ± 0.5 (88.1-89.2)	(89.8, 90.3)	89.8 ± 0.5 (89.2-90.4)	88.7 ± 0.1 (88.6-88.9)	89.5 ± 0.7 (88.6-90.0)	89.5 ± 1.3 (87.7-91.3)	88.1 ± 0.4 (87.6-88.6)
VL/VB	1.3 ± 0.1 (1.1-1.4)	(1.2, 1.3)	1.1 ± 0.2 (1.0-1.4)	1.4 ± 0.1 (1.3-1.4)	1.2 ± 0.1 (1.1-1.3)	1.3 ± 0.1 (1.1-1.4)	1.3 ± 0.1 (1.1-1.4)
Stylet	94.4 ± 3.8 (89.0-98.0)	(90.0, 91.0)	93.2 ± 3.2 (89.0-89.0)	96.8 ± 2.3 (94.0-99.0)	88.7 ± 5.4 (80.0-94.0)	98.2 ± 2.7 (94.0-101.0)	98.0 ± 2.5 (94.0-100.0)
Conus	87.0 ± 3.7 (82.0-91.0)	(82.0, 84.0)	86.0 ± 2.9 (82.0-89.0)	89.4 ± 2.1 (87.0-92.0)	81.4 ± 5.8 (72.0-87.0)	90.6 ± 2.5 (87.0-93.0)	90.8 ± 2.7 (87.0-93.0)
Pharynx	136.4 ± 6.6 (130-147)	(120, 119)	135.6 ± 3.9 (131-141)	130.4 ± 7.5 (122-142)	118.4 ± 10.3 (102-128)	133.0 ± 9.1 (123-146)	135.2 ± 2.6 (132-138)
Max. body width	30.6 ± 6.0 (26.0-41.0)	(29.0, 28.0)	36.0 ± 6.0 (28.0-44.0)	31.8 ± 3.1 (29.0-37.0)	28.4 ± 1.6 (26.5-31.0)	33.0 ± 5.6 (27.0-40.0)	31.6 ± 4.2 (29.0-39.0)
Anal body diam.	20.0 ± 2.7 (17.0-24.0)	(21.0, 21.0)	22.4 ± 2.7 (20.0-27.0)	23.6 ± 3.8 (19.5-29.0)	19.4 ± 1.1 (18.0-21.0)	20.7 ± 2.2 (17.0-22.0)	20.8 ± 0.8 (20.0-22.0)
Vulva to anus distance	15.0 ± 0.7 (14.0-16.0)	(14.0, 15.0)	15.9 ± 0.9 (15.0-17.0)	15.5 ± 1.1 (14.0-17.0)	14.0 ± 1.9 (12.0-17.0)	17.6 ± 1.1 (16.0-19.0)	16.0 ± 0.7 (15.0-17.0)
Tail	18.2 ± 1.6 (17.0-20.0)	(15.0, 16.0)	17.5 ± 3.3 (13.0-22.0)	18.9 ± 2.3 (16.0-22.0)	15.2 ± 0.8 (14.0-16.0)	16.9 ± 1.7 (16.0-20.0)	18.6 ± 1.5 (17.0-21.0)

^a^Measurements are in µm and in the form: (mean) ± (standard deviation), (range). (-) Not obtained or not performed

^b^See Table 1 for identifying the sampled localities

#### Holotype

Adult female collected from a soil sample from the rhizosphere of Pyrenean oak (*Quercus pyrenaica* Willd.) at Cotillas, Albacete province, southern Spain (38°24′24.15″N, 2°28′36.72″W, 1533 m above sea level) by P. Castillo, mounted in pure glycerine, and deposited in the Nematode Collection of the Institute for Sustainable Agriculture, CSIC, Córdoba, Spain (slide number Xen_cot_01).

#### Paratypes

Eighteen female paratypes were collected at the same time as the holotype from the type locality by P. Castillo, mounted in pure glycerine and deposited in the Nematode Collection of the Institute for Sustainable Agriculture, CSIC, Córdoba, Spain (slides numbers Xen_cot_02-Xen_cot_9), and two females were deposited at the USDA Nematode Collection (slide T-8025p).

#### Etymology

The specific epithet is named for the wide distribution of the species in the Iberian peninsula.

#### Diagnosis and relationships

*Xenocriconemella iberica* sp. nov. is characterized by the following measurements and ratios (considering all the studied populations, Tables 3, 4): a short-sized female body 246-350 µm, stylet = 80.0-103.0 µm long, V = 87.5–91.9, a = 6.4–11.9, b = 2.0–2.7, c = 12.1–27.3, c’ = 0.6–1.1, R = 97-119, RV = 11–16, Ran = 7–12, VL/VB = 0.7–1.4. Morphologically and morphometrically, *X. iberica* sp. nov. resembles members of the *X. macrodora* species complex (including *X. macrodora*, *X. paraiberica* sp. nov. and *X. pradense* sp. nov.) from which it is very difficult to separate; in particular, it is almost undistinguishable phenotypically from *X. macrodora* and *X. paraiberica* sp. nov. (Tables 3, 4, 5, 6 and 7, Table S1). From *X. pradense* sp. nov. slightly differs in some main diagnostic characters, including a slightly shorter body length 294 (246–350) µm *vs* 333 (249–383) µm, a slightly shorter stylet length 93.1 (80.0–103.0) µm *vs* 101.1 (92.5–110.0) µm, a slightly lower number of body annuli (R) 104 (97–119) *vs* 122 (112–128), a slightly lower VL/VB ratio 1.2 (0.7–1.4) *vs* 1.4 (1.1–1.5), a slightly shorter tail length 16.4 (11.0–24.5) µm *vs* 20.2 (15.5–25.0) µm, a slightly higher c ratio 18.3 (12.1–27.3) *vs* 16.6 (13.7–21.3), and a slightly lower c’ ratio 0.8 (0.6–1.1) *vs* 0.9 (0.8–1.2). In any case, these minor differences are within the range of the *X. macrodora* species complex, and all four species need to be considered as a complex of cryptic species (see above morphometric study).

**Table 5:** Morphometrics of *Xenocriconemella paraiberica* sp. nov. from the rhizosphere of forest trees in several localities in Spain

**Character/Ratio**^**a**^	**Holotype**	**Paratype Females**	**Females**	**Females**	**Females**	**Females**	**Females**	**Females**
Sample code^b^	CAS22	CAS22	CZQ05	CZE26	COA01	CAC01	CNR01	HUE00
n	1	20	4	5	4	5	4	5
L	292	291.5 ± 16.3 (251-325)	276.8 ± 20.1 (260-303)	309.2 ± 18.2 (293-336)	290.0 ± 21.5 (266-318)	289.6 ± 16.5 (263-306)	273.3 ± 24.3 (239-294)	300.8 ± 14.3 (292-326)
R	106	104.2 ± 3.5 (98-113)	101.0 ± 8.2 (95-113)	101.8 ± 2.2 (100-105)	103.3 ± 2.2 (101-106)	102.4 ± 4.2 (97-106)	101.8 ± 3.0 (98-105)	101.4 ± 2.3 (98-104)
Rst	35	32.6 ± 1.9 (29-36)	33.8 ± 2.6 (30-36)	33.6 ± 2.1 (31-36)	33.8 ± 1.0 (33-35)	34.6 ± 1.1 (33-36)	33.8 ± 1.7 (32-36)	33.8 ± 1.3 (32-35)
Roes	46	42.5 ± 3.4 (38-48)	45.3 ± 5.6 (37-49)	43.2 ± 3.7 (39-48)	44.3 ± 1.0 (43-45)	44.8 ± 1.3 (43-46)	45.8 ± 1.7 (44-48)	43.4 ± 2.2 (40-46)
Rex	36	33.7 ± 1.8 (38-48)	35.5 ± 3.1 (31-38)	34.2 ± 1.6 (32-36)	35.5 ± 1.3 (34-37)	36.2 ± 0.8 (35-37)	34.8 ± 1.7 (33-37)	34.8 ± 1.3 (33-36)
RV	13	11.6 ± 0.7 (11-13)	12.0 ± 0.8 (11-13)	12.2 ± 0.4 (12-13)	12.0 ± 0.8 (11-13)	12.6 ± 0.9 (12-14)	12.0 ± 0.8 (11-13)	12.4 ± 1.1 (11-14)
Rvan	4	4.1 ± 0.3 (4-5)	4.0 ± 0.0 (4-4)	4.0 ± 0.0 (4-4)	4.0 ± 0.0 (4-4)	4.4 ± 0.5 (4-5)	4.0 ± 0.0 (4-4)	4.2 ± 0.4 (4-5)
Ran	9	7.6 ± 0.8 (7-9)	8.0 ± 0.8 (7-9)	8.2 ± 0.4 (8-9)	8.3 ± 1.0 (7-9)	8.2 ± 0.4 (8-9)	8.0 ± 0.8 (7-9)	8.2 ± 0.8 (7-9)
O	8.0	10.4 ± 0.9 (9.0-11.6)	6.8 ± 0.7 (6.0-7.6)	7.8 ± 0.8 (6.5-8.7)	8.7 ± 1.1 (7.9-9.5)	7.8 ± 1.3 (6.5-9.1)	8.8 ± 1.4 (7.5-10.5)	6.8 ± 1.3 (5.4-8.6)
a	8.8	10.3 ± 0.7 (8.8-11.6)	10.0 ± 1.1 (9.0-11.3)	8.6 ± 0.7 (7.6-9.6)	10.7 ± 0.8 (9.9-11.8)	10.8 ± 0.6 (10.0-11.3)	10.5 ± 0.4 (10.0-10.9)	10.5 ± 0.4 (9.9-10.9)
b	2.6	2.6 ± 0.3 (2.2-3.1)	2.6 ± 0.1 (2.5-2.8)	2.6 ± 0.3 (2.4-3.1)	2.4 ± 0.1 (2.4-2.5)	2.5 ± 0.2 (2.3-2.7)	2.4 ± 0.2 (2.2-2.6)	2.4 ± 0.1 (2.2-2.6)
c	19.5	19.5 ± 2.8 (14.4-24.2)	23.2 ± 2.2 (20.1-25.3)	20.6 ± 2.5 (16.8-23.1)	19.6 ± 2.3 (16.7-21.9)	20.2 ± 3.4 (16.1-25.2)	22.4 ± 2.6 (19.9-26.0)	18.3 ± 1.4 (16.3-19.9)
c’	0.9	0.8 ± 0.06 (0.7-0.9)	0.8 ± 0.05 (0.8-0.9)	0.7 ± 0.08 (0.7-0.9)	0.8 ± 0.05 (0.8-0.9)	0.8 ± 0.08 (0.7-0.9)	0.8 ± 0.03 (0.7-0.8)	0.8 ± 0.08 (0.7-1.0)
V or T	89.0	90.1 ± 0.8 (88.8-91.7)	90.1 ± 0.7 (89.2-90.8)	90.5 ± 0.6 (89.8-91.2)	89.9 ± 0.7 (89.1-90.7)	90.0 ± 1.2 (88.2-91.3)	90.0 ± 1.1 (89.1-91.6)	88.6 ± 2.3 (84.6-89.9)
VL/VB	1.1	1.1 ± 0.1 (1.0-1.3)	1.0 ± 0.1 (0.8-1.0)	1.0 ± 0.1 (0.9-1.2)	1.3 ± 0.1 (1.3-1.4)	1.2 ± 0.1 (1.1-1.3)	1.1 ± 0.1 (1.0-1.3)	1.2 ± 0.1 (1.0-1.3)
Stylet	81.0	87.5 ± 3.9 (80.0-94.5)	88.8 ± 5.0 (84.0-94.0)	92.4 ± 1.5 (91.0-95.0)	94.3 ± 4.1 (89.0-99.0)	90.3 ± 3.3 (87.0-94.5)	84.8 ± 3.4 (80.0-88.0)	93.6 ± 1.5 (92.0-96.0)
Conus	75.0	81.1 ± 3.8 (74.0-87.5)	81.5 ± 4.7 (77.0-86.0)	84.8 ± 1.5 (83.0-87.0)	87.5 ± 3.7 (83.0-92.0)	83.9 ± 3.2 (80.0-87.5)	78.3 ± 3.3 (74.0-82.0)	86.2 ± 1.3 (85.0-88.0)
Pharynx	113.0	114.6 ± 11.9 (93-138)	105.8 ± 3.1 (103-110)	120.8 ± 14.2 (104-142)	118.5 ± 6.6 (110-126)	115.4 ± 10.5 (105-133)	112.3 ± 2.9 (109-116)	127.0 ± 3.5 (124-132)
Max. body width	33.0	28.6 ± 2.5 (25.0-33.0)	27.8 ± 1.9 (25.0-29.0)	36.1 ± 4.8 (31.0-44.0)	27.0 ± 0.8 (26.0-28.0)	26.8 ± 1.5 (25.0-29.0)	26.0 ± 1.4 (24.0-27.0)	28.8 ± 1.3 (27.0-30.0)
Anal body diam.	17.0	18.3 ± 1.8 (16.0-21.0)	15.0 ± 1.2 (14.0-16.0)	20.9 ± 1.4 (19.0-22.5)	18.0 ± 2.2 (16.0-21.0)	18.2 ± 1.9 (16.0-21.0)	16.3 ± 1.7 (14.0-18.0)	19.7 ± 1.2 (18.5-21.0)
Vulva to anus distance	14.0	11.8 ± 3.3 (9.0-20.0)	10.5 ± 1.3 (9.0-12.0)	12.4 ± 1.6 (10.0-14.0)	12.0 ± 1.6 (10.0-14.0)	12.0 ± 2.2 (9.0-14.0)	13.3 ± 1.3 (12.0-15.0)	14.1 ± 0.5 (13.5-15.0)
Tail	15.0	15.2 ± 2.5 (25.0-33.0)	12.0 ± 1.4 (11.0-14.0)	15.2 ± 2.3 (13.0-19.0)	15.0 ± 2.7 (13.0-19.0)	14.7 ± 2.7 (11.5-19.0)	12.3 ± 1.0 (11.0-13.0)	16.5 ± 2.0 (15.0-20.0)

^a^Measurements are in µm and in the form: (mean) ± (standard deviation), (range). (-) Not obtained or not performed

^b^See Table 1 for identifying the sampled localities

**Table 6:** Morphometrics of *Xenocriconemella paraiberica* sp. nov. from the rhizosphere of forest trees in several localities in Spain

**Character/Ratio**^**a**^	**Females**	**Females**	**Females**	**Females**	**Females**	**Females**
Sample code^b^	HUA03	BUQ01	GUR04	GUR03	XN43A	XN55A
n	5	4	5	5	4	5
L	329.0± 29.8 (307-381)	242.5 ± 25.2 (221-279)	325.6 ± 21.4 (301-358)	306.2 ± 53.1 (240-386)	323.3 ± 4.4 (319-328)	324.6 ± 21.9 (299-345)
R	102.6 ± 2.9 (98-105)	99.3 ± 3.9 (96-105)	103.0 ± 2.5 (100-106)	104.0 ± 4.7 (100-112)	107.8 ± 1.7 (106-110)	113.0 ± 2.8 (110-116)
Rst	34.0 ± 1.4 (32-36)	36.8 ± 3.4 (32-40)	32.8 ± 1.3 (31-34)	33.8 ± 1.5 (32-36)	35.5 ± 1.3 (34-37)	35.2 ± 0.8 (34-36)
Roes	44.2 ± 2.9 (40-48)	48.3 ± 2.6 (46-52)	42.0 ± 2.3 (39-44)	43.2 ± 1.3 (41-44)	47.3 ± 1.7 (45-49)	47.2 ± 1.3 (46-49)
Rex	35.0 ± 1.4 (33-37)	39.5 ± 3.1 (37-44)	33.8 ± 1.3 (32-35)	34.4 ± 0.9 (33-35)	35.5 ± 2.1 (33-38)	36.4 ± 0.9 (35-37)
RV	12.0 ± 0.7 (11-13)	11.8 ± 1.0 (11-13)	12.0 ± 1.0 (11-13)	11.4 ± 0.5 (11-12)	12.3 ± 0.5 (12-13)	13.0 ± 1.0 (12-14)
Rvan	4.0 ± 0.0 (4-4)	4.0 ± 0.0 (4-4)	4.0 ± 0.0 (4-4)	4.0 ± 0.0 (4-4)	4.0 ± 0.0 (4-4)	4.0 ± 0.0 (4-4)
Ran	8.0 ± 0.7 (7-9)	7.8 ± 1.0 (7-9)	8.0 ± 1.0 (7-9)	7.4 ± 0.5 (7-8)	8.3 ± 0.5 (8-9)	9.0 ± 1.0 (8-10)
O	6.7 ± 1.3 (5.0-8.3)	9.0 ± 0.4 (8.6-9.5)	8.2 ± 0.6 (7.5-8.9)	8.2 ± 1.2 (7.1-9.8)	8.3 ± 0.7 (7.8-9.1)	8.4 ± 0.7 (7.6-9.2)
a	10.1 ± 1.1 (9.2-11.5)	8.5 ± 1.1 (7.4-9.8)	8.8 ± 1.1 (7.7-10.0)	7.8 ± 0.3 (7.4-8.0)	11.4 ± 1.1 (10.3-12.8)	10.9 ± 1.0 (9.1-11.5)
b	2.4 ± 0.3 (2.1-2.8)	2.2 ± 0.1 (2.2-2.4)	2.4 ± 0.1 (2.3-2.6)	2.3 ± 0.2 (2.1-2.5)	2.4 ± 0.1 (2.3-2.5)	2.6 ± 0.2 (2.3-2.8)
c	19.6 ± 2.6 (16.2-23.0)	17.6 ± 4.8 (13.0-23.6)	20.0 ± 2.9 (16.7-23.0)	23.0 ± 3.4 (19.5-28.6)	20.8 ± 2.2 (18.2-23.3)	19.9 ± 2.5 (16.8-22.3)
c’	0.8 ± 0.14 (0.6-1.0)	0.8 ± 0.09 (0.7-0.9)	0.7 ± 0.13 (0.6-0.9)	0.7 ± 0.08 (0.6-0.8)	0.8 ± 0.07 (0.7-0.8)	0.9 ± 0.09 (0.8-1.0)
V or T	90.2 ± 0.3 (89.9-90.7)	90.2 ± 0.4 (89.8-90.6)	89.5 ± 0.5 (88.6-90.1)	90.1 ± 0.6 (89.4-90.9)	90.2 ± 0.3 (89.9-90.5)	89.7 ± 0.7 (88.9-90.4)
VL/VB	1.1 ± 0.2 (0.9-1.3)	1.0 ± 0.1 (0.8-1.1)	1.0 ± 0.2 (0.8-1.2)	0.8 ± 0.1 (0.7-0.9)	1.3 ± 0.1 (1.2-1.4)	1.3 ± 0.2 (1.0-1.5)
Stylet	96.4 ± 2.5 (93.0-100.0)	83.6 ± 3.2 (81.0-88.0)	92.2 ± 2.0 (90.0-94.0)	90.5 ± 6.5 (82.0-99.0)	88.8 ± 1.5 (87.0-90.0)	86.8 ± 4.3 (82.0-92.0)
Conus	86.8 ± 2.9 (82.0-89.0)	76.3 ± 3.3 (74.0-81.0)	84.4 ± 1.8 (82.0-86.0)	82.8 ± 6.1 (75.0-91.0)	81.5 ± 1.3 (80.0-83.0)	79.6 ± 4.0 (75.0-84.0)
Pharynx	136.8 ± 11.7 (124-156)	108.5 ± 6.8 (102-118)	133.8 ± 13.9 (120-153)	134.0 ± 13.3 (115-152)	133.0 ± 5.4 (128-139)	124.6 ± 3.8 (120-129)
Max. body width	32.9 ± 5.6 (28.0-41.5)	28.6 ± 1.9 (26.0-30.0)	37.5 ± 5.5 (32.0-43.0)	39.4 ± 6.5 (30.0-48.0)	28.5 ± 3.1 (25.0-32.0)	30.1 ± 4.8 (26.0-38.0)
Anal body diam.	21.1 ± 1.5 (19.0-23.0)	18.8 ± 3.4 (14.0-22.0)	22.6 ± 2.5 (19.5-26.0)	20.3 ± 4.6 (15.0-26.5)	18.8 ± 1.0 (18.0-20.0)	18.2 ± 1.6 (16.0-20.0)
Vulva to anus distance	15.4 ± 0.9 (15.0-17.0)	12.4 ± 1.3 (11.0-14.0)	15.4 ± 1.8 (13.0-17.0)	14.1 ± 1.1 (13.0-16.0)	15.0 ± 1.7 (14.0-17.0)	17.6 ± 2.8 (15.0-21.0)
Tail	17.0 ± 2.5 (14.0-20.0)	14.4 ± 3.1 (10.0-17.0)	16.6 ± 3.2 (14.0-21.0)	13.6 ± 3.4 (10.0-18.0)	15.6 ± 1.5 (14.0-17.5)	16.5 ± 1.9 (15.0-19.0)

^a^Measurements are in µm and in the form: (mean) ± (standard deviation), (range). (-) Not obtained or not performed

^b^See Table 1 for identifying the sampled localities

**Table 7:** Morphometrics of *Xenocriconemella pradense* sp. nov. from the rhizosphere of Portuguese oak forest trees (*Quercus faginea* Lam.) in Prado del Rey, Cádiz, Spain

**Character/Ratio**^**a**^	**Holotype**	**Female Paratypes**	**Females**	**Females**
Sample code^b^	GRQ01	GRQ01	GRQ02	GRQ05
n	1	20	5	5
L	327	327.9 ± 26.5 (249-375)	330.8 ± 32.2 (300-383)	353.6 ± 10.5 (342-367)
R	122	121.1 ± 3.6 (112-128)	122.0 ± 1.6 (120-124)	125.4 ± 2.1 (123-128)
Rst	40	41.2 ± 2.1 (37-46)	40.8 ± 2.6 (38-44)	43.0 ± 3.5 (40-48)
Roes	56	57.1 ± 2.9 (49-62)	57.6 ± 1.1 (56-59)	53.2 ± 3.8 (50-59)
Rex	41	41.3 ± 2.4 (38-47)	40.8 ± 1.5 (39-43)	44.0 ± 3.5 (41-49)
RV	15	15.9 ± 0.7 (15-18)	15.2 ± 0.8 (14-16)	15.4 ± 0.5 (15-16)
Rvan	4	4.4 ± 0.5 (4-5)	4.2 ± 0.4 (4-5)	4.0 ± 0.0 (4-4)
Ran	11	11.6 ± 0.6 (11-13)	11.0 ± 0.7 (10-12)	11.4 ± 0.5 (11-12)
O	8.0	7.7 ± 0.7 (6.6-8.9)	7.4 ± 0.6 (6.6-8.2)	7.6 ± 0.3 (7.2-8.1)
a	11.3	9.6 ± 1.4 (7.5-12.3)	11.7 ± 0.7 (10.8-12.8)	10.1 ± 0.7 (9.1-10.9)
b	2.2	2.4 ± 0.2 (1.8-2.7)	2.4 ± 0.2 (2.2-2.7)	2.4 ± 0.2 (2.2-2.6)
c	17.2	16.6 ± 1.8 (13.7-19.7)	17.3 ± 2.7 (14.9-21.3)	15.9 ± 1.1 (14.3-17.1)
c’	1.1	0.9 ± 0.1 (0.8-1.1)	0.9 ± 0.1 (0.8-1.1)	1.1 ± 0.1 (0.7-1.2)
V or T	88.4	88.3 ± 1.2 (85.7-90.2)	88.3 ± 0.9 (87.2-89.4)	89.0 ± 0.6 (88.3-90.0)
VL/VB	1.5	1.3 ± 0.1 (1.1-1.5)	1.4 ± 0.1 (1.3-1.5)	1.4 ± 0.1 (1.3-1.5)
Stylet	100.0	100.5 ± 4.8 (92.5-110.0)	103.4 ± 3.8 (98.0-107.0)	101.0 ± 3.4 (97.0-105.0)
Conus	92.0	92.3 ± 4.1 (85.0-102.0)	95.0 ± 3.1 (90.0-97.0)	93.2 ± 3.1 (90.0-97.0)
Pharynx	148.0	138.5 ± 8.6 (122-156)	137.6 ± 8.7 (128-148)	148.2 ± 14.2 (132-162)
Max. body width	29.0	34.9 ± 5.6 (28.0-45.0)	28.2 ± 1.9 (25.0-30.0)	35.1 ± 2.9 (35.1-39.0)
Anal body diam.	18.0	21.7 ± 3.1 (14.5-28.0)	21.0 ± 2.7 (18.0-25.0)	21.3 ± 1.5 (19.5-23.0)
Vulva to anus distance	17.0	17.7 ± 2.0 (13.0-21.0)	18.4 ± 3.0 (14.0-22.0)	15.9 ± 1.8 (13.0-18.0)
Tail	19.0	19.9 ± 2.4 (15.5-25.0)	19.3 ± 2.2 (16.0-21.0)	22.3 ± 1.2 (21.0-24.0)

^a^Measurements are in µm and in the form: (mean) ± (standard deviation), (range). (-) Not obtained or not performed

^b^See Table 1 for identifying the sampled localities

#### Description

*Female*. Nematodes ventrally arcuate, slightly tapering anteriorly and posteriorly. Body annuli smooth and retrorse 2.7 (2.5–3.0) µm wide, without anastomosis. Lip region with two annuli, not offset, not separated from body annuli, first lip annulus partially covering the second lip annulus (Fig. 9b), second lip annulus retrorse and slightly wider than first annulus (8.1 ± 0.7 (7.0–9.0) *vs* (6.8 ± 0.5 (6.0–8.0)) µm wide. SEM images (Fig. 11) showed a labial plate low, with oral aperture oval, pseudolips not visible, and submedian lobes absent. Stylet thin, long and flexible, occupying 32.5 (28.1–35.6) % of the body length, with short basal portion (6.1 (5.0–7.5)) µm long, and knobs slightly rounded (4.5 (3.5–5.0)) µm wide. Pharynx typical criconematoid, with a cylindroid procorpus widening to a large muscular oval median bulb containing well developed valves (7.0–8.5 µm long), isthmus slender and amalgamated with basal bulb. Excretory pore from one annulus posterior to three annuli anterior of level of stylet knobs, at 92 (82–106) µm from anterior end. Nerve ring located at level of isthmus, 100 (91–113) µm from anterior end. SEM showed a vulva closed as a simple slit, directed out of the contour of the body (Fig. 11e), with the anterior vulval lip nonoverlapping. Vagina slightly ventrally curved (11–14 µm long). Female genital tract monodelphic, prodelphic, outstretched and occupying 46.1 (38.8–58.6) % of the body length, spermatheca rounded, some females (*ca*. 30%) containing round sperm (1.0-1.5 µm wide). Anus located at 8.6 (7–9) annuli from the terminus. Tail conoid and bluntly rounded terminus, annuli decreasing in diameter and thickness, the last 2–3 annuli merging and difficult to count.

*Male*. Nematodes extremely rare, with only two specimens detected in a sample from Aracena, Huelva Province, southern Spain, out of 4527 female and juvenile specimens counted within the 13 populations studied (Table 1). Both male specimens were sequenced for ribosomal and mitochondrial genes, confirming their species identity. Body slightly curved ventrally, narrowing to the tail region (Fig. 10). The lip region was conoid-rounded, the stylet was absent, the pharynx was undistinguishable and not functional, lateral fields with three incisures observed. The testis was straight and it was 38.8, 68.1% of the total body length. Tail conoid with a widely rounded terminus. Bursa small (28–29 µm) extending from the posterior third of spicules to the terminus. Spicules slender and ventrally curved, gubernaculum rod-shaped and slightly curved ventrally (Fig. 10).

*Juveniles*. Body similar to females, including tail shape, but shorter. Edge of body annuli without appendages, marked with delicate irregular punctations.

*Additional material studied*. Additional populations of this species were collected from several localities in Portugal and Spain from the rhizosphere of *Castanea sativa* L., *Fagus sylvatica* L., *Quercus canariensis* Willd., *Quercus faginea* Lam., *Quercus ilex* L., *Quercus pubescens* Willd., *Quercus pyrenaica* Willd., *Quercus suber* L. (Table 1).

### *Xenocriconemella paraiberica* sp. nov. (Figs. 12, 13 and 14; Tables 5, 6)

#### Zoobank

**Fig. 12:**
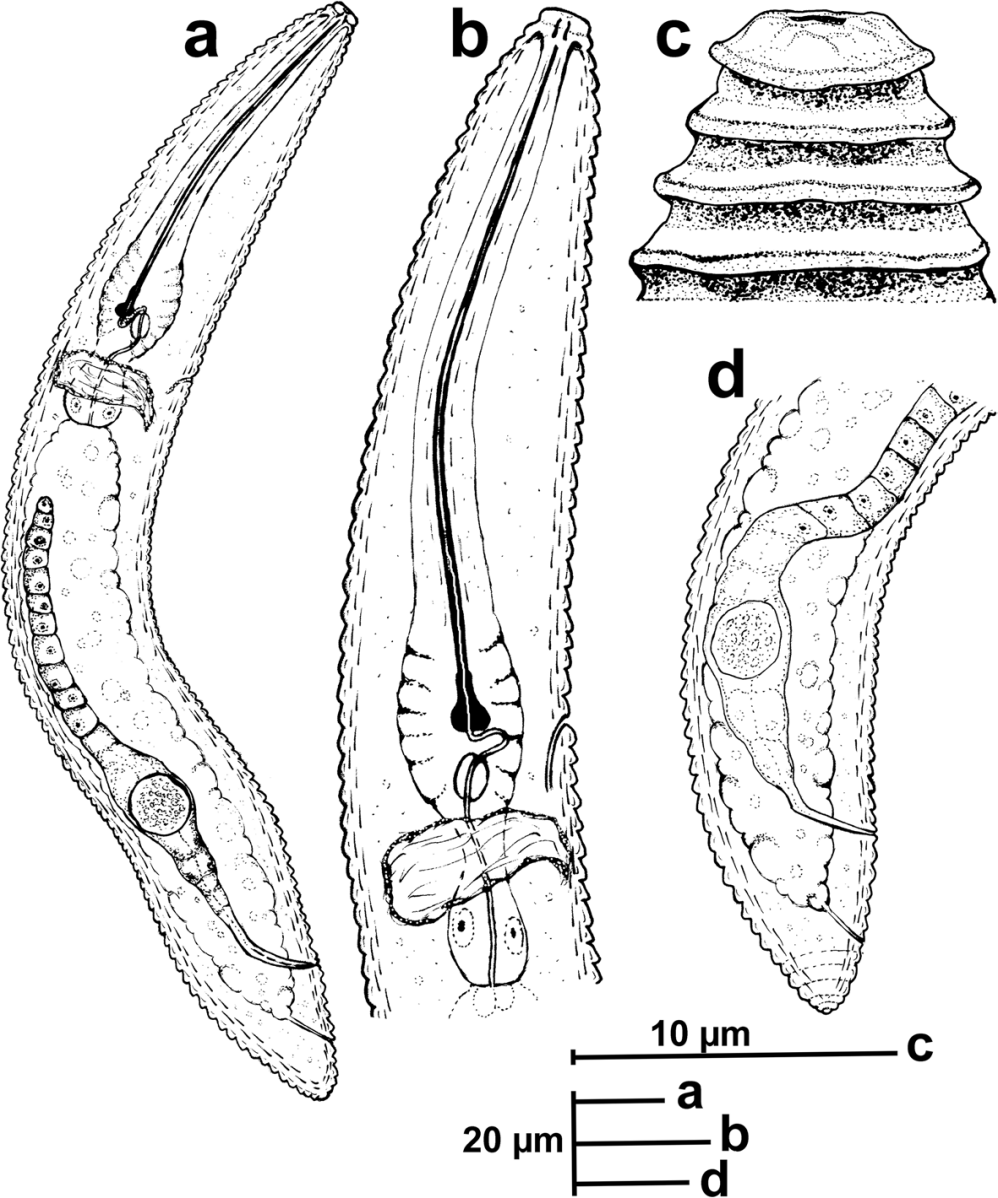
Line drawings of *Xenocriconemella paraiberica* sp. nov. **a** whole female: (**b**) female anterior region; (**c**) lip region showing details of the 1^st^ and 2^nd^ annuli; (**d**) female posterior region

**Fig. 13:**
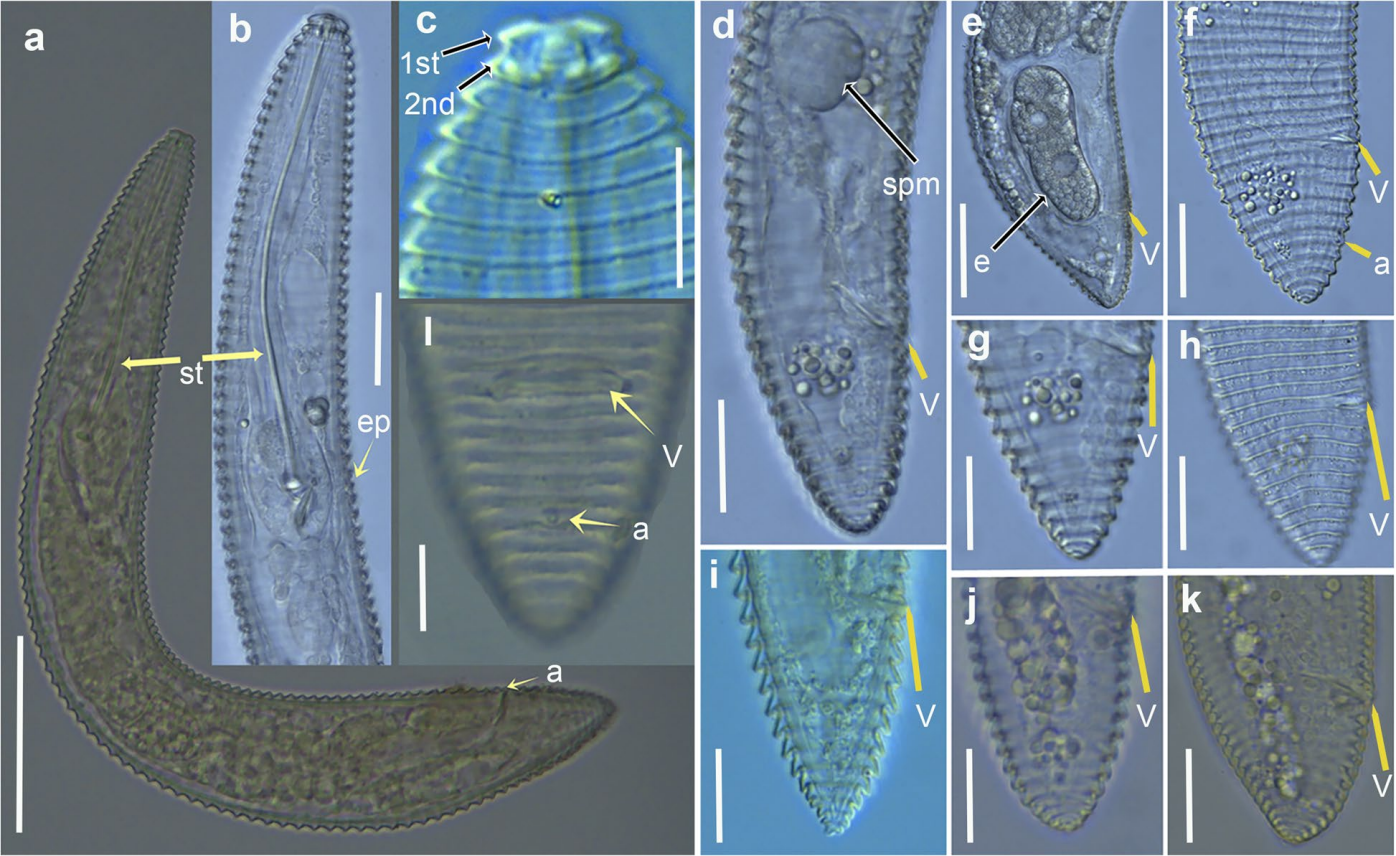
Light micrographs of *Xenocriconemella paraiberica* sp. nov. females. **a**, whole body, flexible stylet arrowed; (**b**), pharyngeal region showing flexible stylet and excretory pore (arrowed); (**c**) lip region showing details of 1st and 2nd body annuli (arrowed); (**d**) posterior region showing spermatheca (arrowed); (**e**-**k**) posterior region showing an egg, vulva and anus (arrowed); (**l**) ventral view of female posterior region showing vulva and anus (arrowed). Abbreviations: a = anus; e = egg; ep = excretory pore; spm = spermatheca, st = stylet; V = vulva; 1st, 2nd = first- and second-body annuli. Scale bars: (**a**) = 50 µm; (**b**–**k**) = 20 µm; (**c**, **l**) = 10 µm

**Fig. 14:**
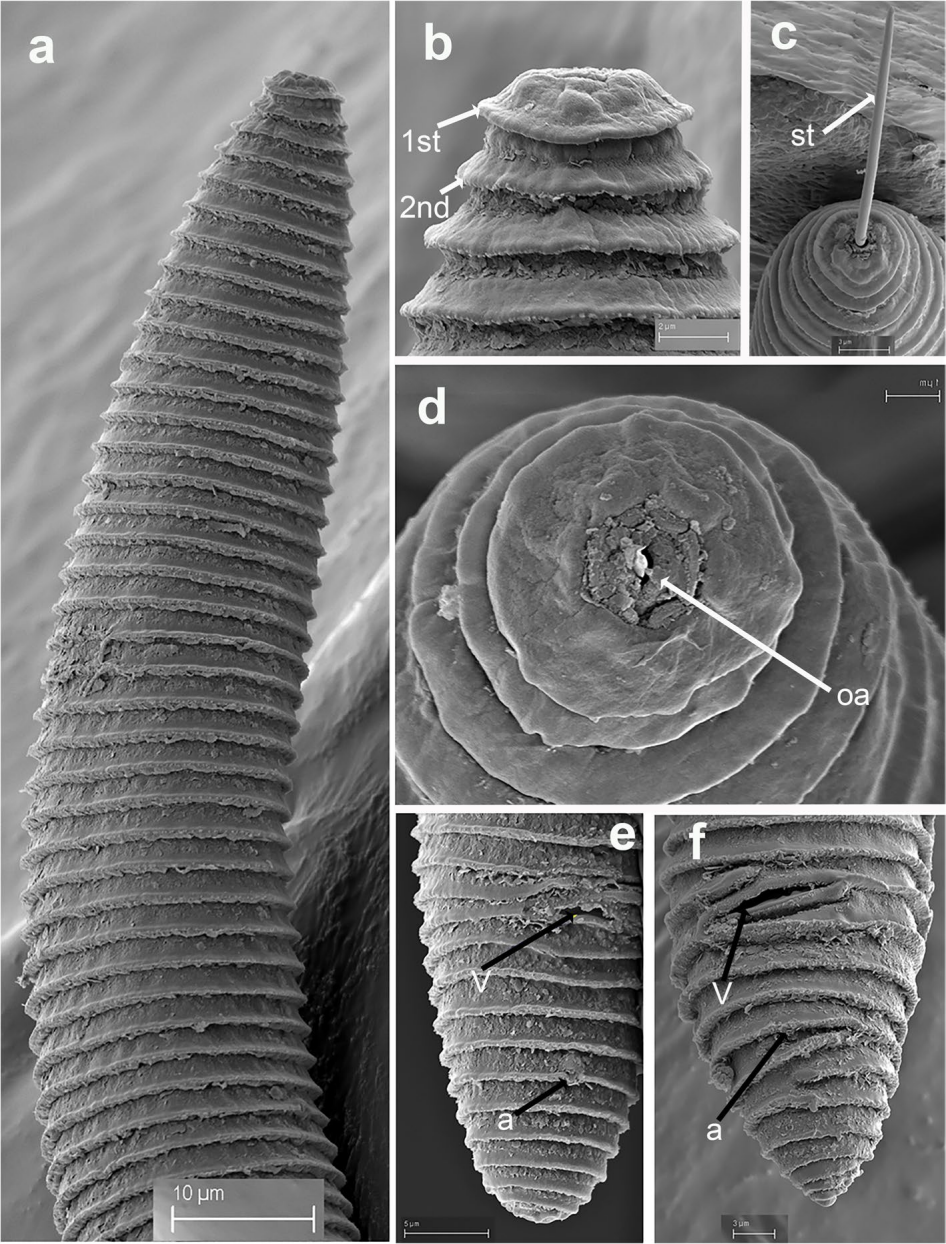
SEM micrographs of *Xenocriconemella paraiberica* sp. nov. female. (**a**) anterior region; (**b**, **c**) lip region with detail of 1st and 2nd body annuli and stylet (arrowed); (**d**) en face view showing oral aperture (arrowed); (**e**, **f**) posterior region in frontal view showing vulva and anus (arrowed). Abbreviations: a = anus; oa = oral aperture; st = stylet; V = vulva; 1st, 2nd = first- and second-body annuli. Scale bars: (**a**) = 10 µm; (**b**) = 2 µm; (**c**, **f**) = 3 µm; (**d**) = 1 µm; (**e**) = 5 µm

urn:lsid:zoobank.org:act:F05DD1CA-662B-46E6-BEB9-377D70788A6C Figs. 12, 13 and 14; Tables 5, 6.

#### Holotype

Adult female collected from a soil sample from the rhizosphere of cork oak (*Quercus suber* L.) at Casares, Málaga Province, southern Spain (36°26′46.21″N, 5°14′57.00″W, 348 m above sea level) by G. Leon Ropero and J. Martin Barbarroja (IAS-CSIC), mounted in pure glycerine and deposited in the Nematode Collection of the Institute for Sustainable Agriculture, CSIC, Córdoba, Spain (slide number Xen_cas_01).

#### Paratypes

Eighteen female paratypes were collected at the same time as the holotype from the type locality by G. Leon Ropero and J. Martin Barbarroja (IAS-CSIC), mounted in pure glycerine and deposited in the Nematode Collection of the Institute for Sustainable Agriculture, CSIC, Córdoba, Spain (slides numbers Xen_cas_02-Xen_cas_11), and two females were deposited at the USDA Nematode Collection (slide T-8026p).

#### Etymology

The specific epithet refers to Gr. prep. para, alongside of, resembling; N.L. fem. n. *iberica*, because of its close resemblance to *Xenocriconemella iberica* sp. nov.

#### Diagnosis and relationships

*Xenocriconemella paraiberica* sp. nov. is characterized by the following measurements and ratios (considering all the studied populations, Tables 5 and 6): a short-sized female body 221–386 µm, stylet = 80.0–100.0 µm long, V = 84.6–91.7, a = 7.4–12.8, b = 2.1–3.1, c = 13.0–28.6, c’ = 0.6–1.0, R = 95–116, RV = 11–14, Ran = 7–10, VL/VB = 0.7–1.5. Morphologically and morphometrically, *X. paraiberica* sp. nov. resembles members of the *X. macrodora* species complex (including *X. macrodora*, *X. iberica* sp. nov. and *X. pradense* sp. nov.) from which it is very difficult to separate it; in particular, it is almost undistinguishable phenotypically from *X. macrodora* and *X. iberica* sp. nov. (Tables 3, 4, 5, 6 and 7, Table S1). From *X. pradense* sp. nov. slightly differs in some main diagnostic characters, including a slightly shorter body length 298 (221–386) µm *vs* 333 (249–383) µm, a slightly shorter stylet length 89.6 (80.0–100.0) µm *vs* 101.1 (92.0–110.0) µm, a slightly lower number of body annuli (R) 104 (95–116) *vs* 122 (112–128), a slightly lower VL/VB ratio 1.1 (0.7–1.5) *vs* 1.4 (1.1–1.5), a slightly shorter tail length 15.1 (10.0–21.0) µm *vs* 20.2 (15.5–25.0) µm, a slightly higher c ratio 20.2 (13.0–28.6) *vs* 16.6 (13.7–21.3), and a slightly lower c’ ratio 0.8 (0.6–1.0) *vs* 0.9 (0.8–1.2). In any case, these minor differences are within the range of the *X. macrodora* species complex, and all four species need to be considered as a complex of cryptic species (see above morphometric study).

#### Description

*Female*. Nematodes ventrally arcuate, slightly tapering anteriorly and posteriorly. Body annuli smooth and retrorse 2.9 (2.5–3.5) µm wide, without anastomosis. Lip region similar to *X. iberica* sp. nov., second lip annulus retrorse and slightly wider than the first annulus (9.1 ± 0.6 (8.0–10.0) *vs* (7.8 ± 0.4 (7.0–8.5)) µm wide. SEM images showed a labial plate low, without pseudolips or submedian lobes (Fig. 14). Stylet thin, long and flexible, occupying 30.1 (27.4–33.1) % of the body length, with short basal portion (6.3 (5.5–10.0)) µm long, and knobs slightly rounded (4.8 (4.0–5.0)) µm wide. Pharynx with a cylindroid procorpus widening to a large muscular oval median bulb containing well-developed valves (8.0–9.0 µm long), istmus slender and amalgamated with basal bulb. Excretory pore from one annulus posterior to the level of stylet knobs, 90 (76–99) µm from the anterior end. Nerve ring located at the level of isthmus, 98 (85–110) µm from the anterior end. Vulva closed as a simple slit, directed out of the contour of the body (Fig. 14e,f), and the anterior vulval lip non-overlapping. Vagina slightly ventrally curved (10–13 µm long). Female genital tract monodelphic, prodelphic, outstretched and occupying 48.8 (32.5–63.7) % of the body length, spermatheca rounded, some females (*ca*. 30%) containing round sperm (1.0–1.5 µm wide). Anus located at 7.6 (7–9) annuli from the terminus. Tail conoid and bluntly rounded terminus, annuli decreasing in diameter and thickness, in some specimens the last 2–3 annuli merging and difficult to count.

*Male*. Not detected in 4046 female and juvenile specimens counted within the 12 populations studied (Table 1).

*Juveniles*. Body similar to females, including tail shape, but shorter. Edge of body annuli without appendages, marked with delicate irregular punctations.

*Additional material examined*. Additional populations of this species were collected from several localities in Spain from the rhizosphere of *Quercus canariensis* Willd., *Quercus faginea* Lam., *Quercus ilex* L., *Quercus pyrenaica* Willd., *Quercus suber* L. (Table 1).

### *Xenocriconemella pradense* sp. nov. (Figs. 15, 16, 17 and 18; Table 7)

#### Zoobank

**Fig. 15:**
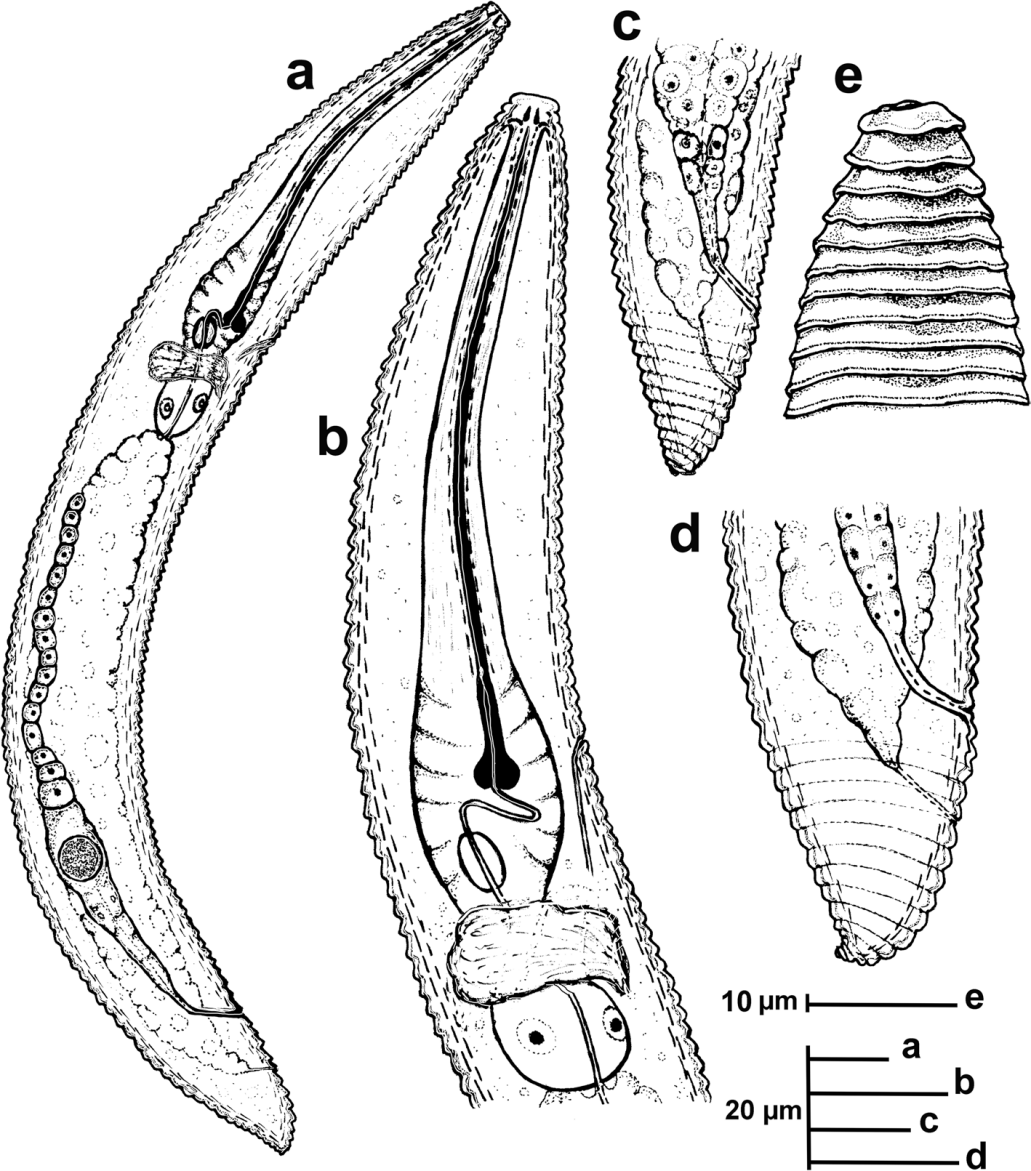
Line drawings of *Xenocriconemella pradense* sp. nov. **a** whole female: (**b**) female anterior region; (**c,** **d**) female posterior region; (**e**) lip region showing details of the 1^st^ and 2^nd^ annuli

**Fig. 16:**
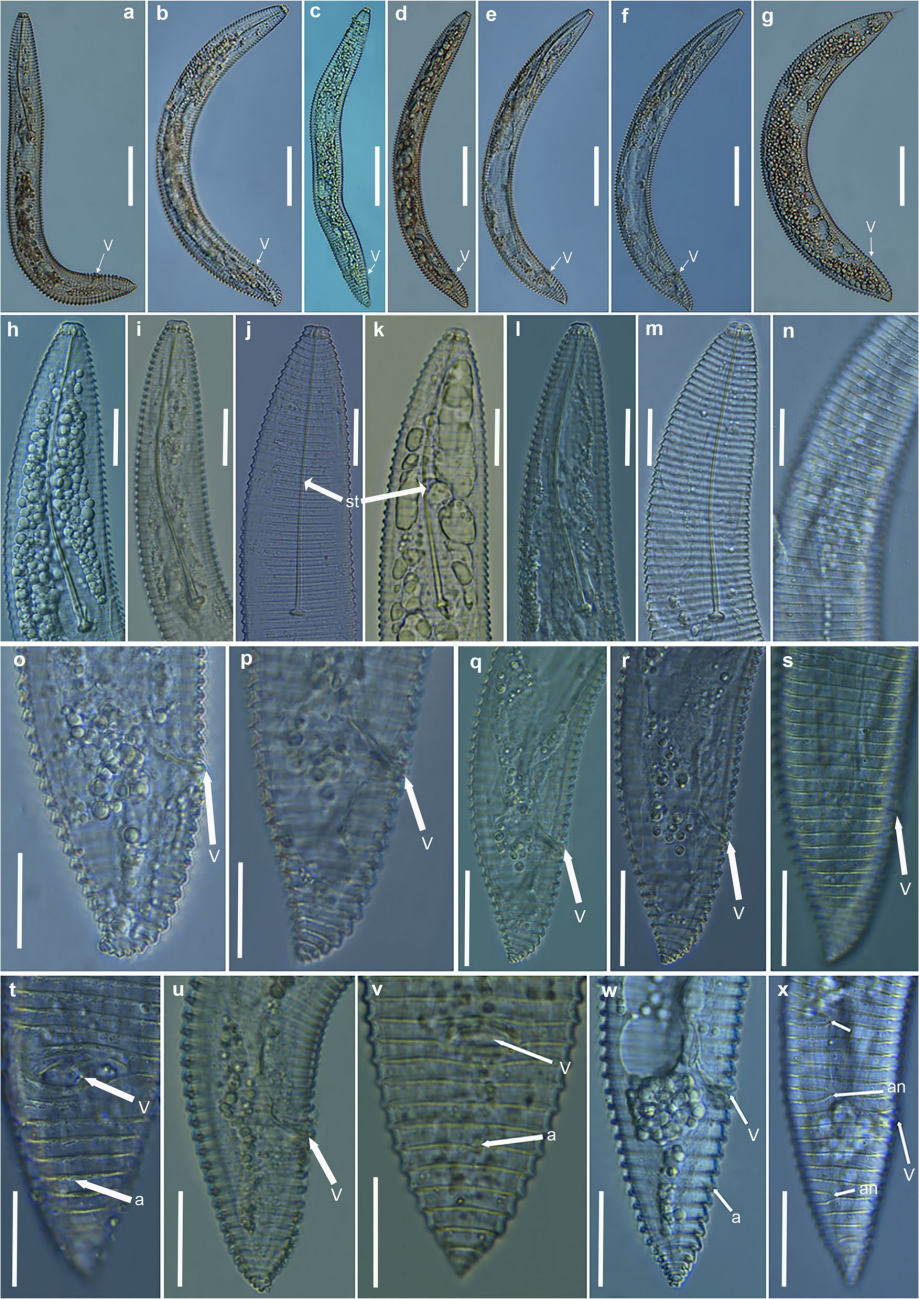
Light micrographs of *Xenocriconemella pradense* sp. nov. females. (**a**–**g**) whole body; (**h**-**n**) anterior region showing flexible stylet (arrowed); (**o**-**x**) posterior region showing anus, vulva, and some anastomosis (arrowed). Abbreviations: a = anus; an = anastomosis; st = stylet; V = vulva. Scale bars: (**a**–**g**) = 50 µm; (**h**–**x**) = 20 µm

**Fig. 17:**
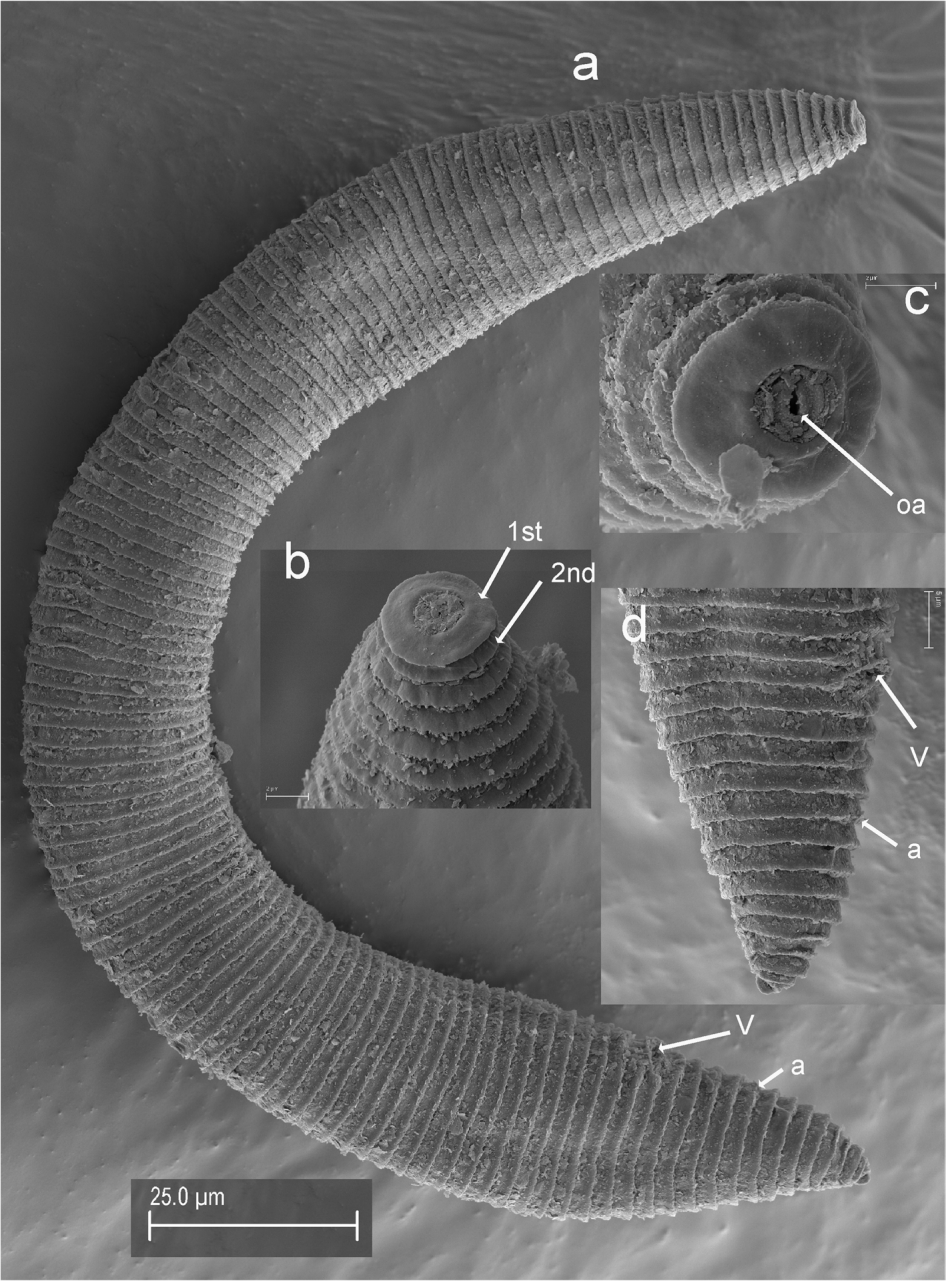
SEM micrographs of *Xenocriconemella pradense* sp. nov. female. **a** whole body; (**b**) detail of 1st and 2nd body annuli; (**c**) en face view showing oral aperture (arrowed); (**d**) posterior region showing vulva and anus (arrowed). Abbreviations: a = anus; oa = oral aperture; V = vulva; 1st, 2nd = first- and second-body annuli. Scale bars: (**a**) = 25 µm; (**b**, **c**) = 2 µm; (**d**) = 5 µm

**Fig. 18:**
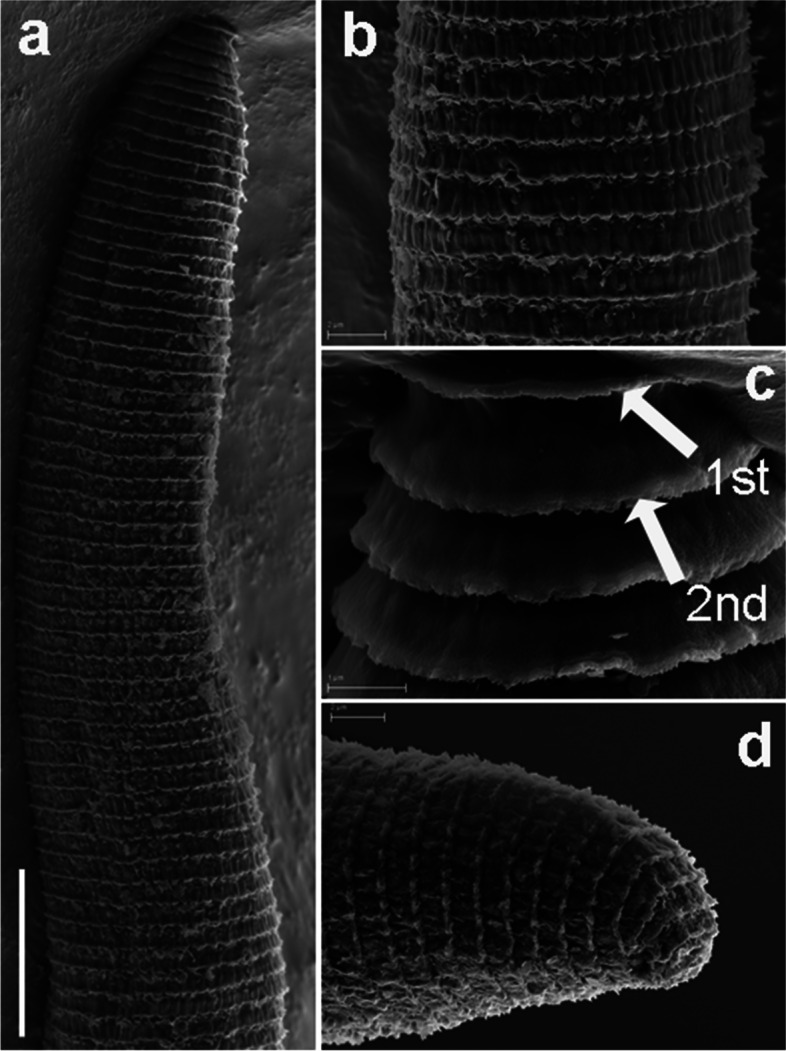
SEM micrographs of *Xenocriconemella pradense* sp. nov. juvenile. **a** anterior region showing ornamented annuli; (**b**) detail of ornamented annuli ant mid-body; (**c**) detail of 1st and 2nd body annuli; (**d**) posterior region showing ornamented annuli. Abbreviations: 1st, 2nd = first- and second-body annuli. Scale bars: (**a**) = 10 µm; (**b**, **d**) = 2 µm; (**c**) = 1 µm

urn:lsid:zoobank.org:act:82EF28F3-3C22-4E8A-9A68-8A63A483F141 Figs. 15, 16, 17 and 18; Table 7.

#### Holotype

Adult female collected from a soil sample from the rhizosphere of Portuguese oak (*Quercus faginea* L.) at Prado del Rey, Cadiz Province, southern Spain (36°45′08.48″N, 5°24′11.18″W, 950 m above sea level) by A. Archidona-Yuste, mounted in pure glycerine and deposited in the Nematode Collection of the Institute for Sustainable Agriculture, CSIC, Córdoba, Spain (slide number Xen_cho_01).

#### Paratypes

Eighteen female paratypes were collected at the same time as the holotype from the type locality by A. Archidona-Yuste, mounted in pure glycerine and deposited in the Nematode Collection of the Institute for Sustainable Agriculture, CSIC, Córdoba, Spain (slides numbers Xen_cho_02-Xen_cho_12), and two females were deposited at the USDA Nematode Collection (slide T-8027p).

#### Etymology

The specific epithet, *pradense*, refers to the demonym of inhabitants of the type locality, Prado del Rey.

#### Diagnosis and relationships

*Xenocriconemella pradense* sp. nov. is characterized by the following measurements and ratios (considering all the studied populations, Table 7): a short-sized female body 249-383 µm, stylet = 92.5–110.0 µm long, V = 85.7–90.2, a = 7.5–12.8, b = 1.8–2.7, c = 13.7–21.3, c’ = 0.8–1.1, R = 112–128, RV = 14–18, Ran = 10–13, VL/VB = 1.1–1.5. Morphologically and morphometrically, *X. pradense* sp. nov. resembles members of the *X. macrodora* species complex (including *X. macrodora*, *X. iberica* sp. nov. and *X. paraiberica* sp. nov.) from which it is very difficult to separate (Tables 3, 4, 5, 6 and 7, Table S1). From *X. iberica* sp. nov. slightly differs in some main diagnostic characters, including a slightly larger body length 333 (249-383) µm *vs* 294 (246-350) µm, a slightly larger stylet length 101.1 (92.5–110.0) µm *vs* 93.1 (80.0–103.0) µm, a slightly higher number of body annuli (R) 122 (112–128) *vs* 104 (97–119), a slightly higher VL/VB ratio 1.4 (1.1–1.5) *vs* 1.2 (0.7–1.4), a slightly larger tail length 20.2 (15.5–25.0) µm *vs* 16.4 (11.0–24.5) µm, a slightly lower c ratio 16.6 (13.7–21.3) *vs* 18.3 (12.1–27.3), and a slightly higher c’ ratio0.9 (0.8–1.1) *vs* 0.8 (0.6–1.1). In any case, these minor differences are within the range of the *X. macrodora* species complex, and all four species need to be considered as a complex of cryptic species (see above morphometric study).

#### Description

*Female*. Nematodes slightly ventrally arcuate, slightly tapering posteriorly. Body annuli smooth and retrorse 2.6 (2.5–3.0) µm wide, without anastomosis. Lip region with two annuli, not offset, not separated from body annuli, first lip annulus partially covering the second lip annulus (Fig. 16), second lip annulus retrorse and slightly wider than first lip annulus (7.9 ± 0.5 (7.0–9.0) *vs* 8.9 ± 0.5 (8.0–10.0)) µm wide. SEM images showed a labial plate low, pseudolips not visible, and submedian lobes absent (Fig. 17c). Stylet thin, long and flexible, occupying 30.6 (26.3–39.0) % of the body length, with short basal portion (8.2 (6.0–11.0)) µm long, and knobs slightly rounded (4.7 (4.0–5.0)) µm wide. Pharynx typical criconematoid, with a cylindroid procorpus widening to a large muscular oval median bulb containing well developed valves (7.5–8.5 µm long), isthmus slender and amalgamated with basal bulb. Excretory pore from one annulus posterior to three annuli anterior of level of stylet knobs, at 101 (80–115) µm from anterior end. Nerve ring located at the level of isthmus, 118 (108–138) µm from the anterior end. Vulva closed as a simple slit, directed out of the contour of the body (Fig. 17d), and the anterior vulval lip non-overlapping. Vagina slightly ventrally curved (12–14 µm long). Female genital tract monodelphic, prodelphic, outstretched and occupying 48.2 (39.4–58.6) % of the body length, spermatheca round-oval (8–9 x 10-11 µm), some females (*ca*. 10%) with round sperm (1.0 µm wide). Anus located at 11.6 (11–13) annuli from the terminus. Tail conoid and bluntly rounded terminus, annuli decreasing in diameter and thickness, the last 2-3 annuli usually merging with terminus into a small projection oriented dorsally (Fig. 16o-x).

*Male*. Not detected in 975 female and juvenile specimens counted within the three populations studied (Table 1).

*Juveniles*. Body similar to females, including tail shape, but shorter. Edge of body annuli without appendages, marked with delicate irregular punctations (Fig. 18).

*Additional material examined*. Two additional populations of this species were collected from other places in the type locality in the rhizosphere Portuguese oak (Table 1).

### SEM remarks on *Criconemoides rosmarini* and the *Xenocriconemella* species complex

SEM studies on female and juvenile topotypes of *Criconemoides rosmarini* (Fig. S1) presented body annuli with margins showing a fringe of small blunt spine-like processes formed by deep incision or crenation, and numerous anastomoses. These crenate processes were also detected in juveniles, which lack spines or scales. First lip annulus forwardly directed, oral plate without submedian lobes, and vulva open type with rounded lips (Fig. S1).

Additionally, in the three *Xenocriconemella* species studied under light microscopy and that on *X. pradense* sp. nov. also with SEM (Fig. 18), the margin of body annuli appeared marked with delicate irregular punctations, except for annuli near the lip region (Fig. 18c), which showed a similar pattern as adult females.

## Discussion

Asystematic review of *X. macrodora* indicated that this species is distributed worldwide and occurs in association with woodland forests [4, 5]. It has been reported widely in USA, and to a lesser extent in Canada and Mexico [5, 10, 49], several European countries (*viz.* Belgium, Bulgaria, Czech Republic, France, Germany, Italy, Netherlands, Norway, Poland, Portugal, Romania, Russia, Slovak Republic, Spain, Ukraine, and United Kingdom) [4, 6, 50–53], South Africa and Malawi [54, 55], several Asian countries including India, Iran, Korea, and Vietnam [14, 56–58], and Australia [59] (Fig. 1). Based on morphological, morphometric and molecular evidence, we found a new cryptic species complex from the nominal species *X. macrodora* in the Iberian peninsula, USA, and probably Italy (this one only based on a ribosomal molecular marker and this can be another different species, but additional studies are needed for confirming this hypothesis). *Xenocriconemella macrodora* species complex is defined here with the additional description of three new species (*X. iberica* sp. nov., *X. paraiberica* sp. nov., and *X. pradense* sp. nov.) applying a broad taxonomic framework on 28 nematode populations (Fig. 1). Our study claimed that the *X. macrodora* species complex is an example of cryptic species, since most of its members can only be recognized using molecular data [60]. More specifically, species delimitation by morphometry showed an overlap between some populations of the new species of *X. macrodora* described here. species complex(*viz. X. iberica* sp. nov., *X. paraiberica* sp. nov., and *X. pradense* sp. nov.) (Fig. 2). However, we certainly support the validity of multivariate analyses in providing a useful tool for species delimitation within cryptic species complex in soil nematodes [15, 18, 61, 62]. Focusing on the three new species, PCA allowed us to discriminate species seemingly undistinguishable morphologically by their morphometric features (i.e. *X. pradense* sp. nov. from the other new species by the body annuli features (R, Rv, Roes and Rex; Fig. 2). In addition, PCA also showed that the populations of these new species are morphometrically described by a rather extensive intraspecific variation (Fig. 2). This study confirms that males are extremely rare within these nematodes (only two specimens were detected in one population of *X. iberica* sp. nov.). Nevertheless, the presence of sperm in the spermatheca of some specimens confirms that parthenogenesis and amphimictic reproduction in these species is highly probable. Apart from their shortage, the difficulty in finding males can also be related to the lack of feeding (stylet absent) and their very short life span. Sturhan [9] found also the presence of filled spermatheca and the males in some *X. macrodora* populations. In our case, these males of *X. iberica* sp. nov are confirmed molecularly as conspecific. This data confirms that males could be produced in some populations, maybe induced in low numbers by environmental cues.

Ribosomal- and mitochondrial-based phylogenies clearly separate the *X. macrodora* species complex into four separate species, which was confirmed by morphometric and molecular species delimitation analyses. All the molecular markers used in the present study match our identified species, clearly separating them from the only species of this genus described to date, *X. macrodora*, giving evidence that they could help in the identification process for the majority of the populations belonging to this genus. As already documented for other ring nematodes [15, 18], the sequence divergence of COI within the *X. macrodora* species complex was higher than that for the D2-D3 and ITS loci, most likely because mtDNA accumulated nucleotide substitutions at a much higher rate of substitutions than D2-D3 and ITS [18]. These COI haplotypes seem to be related to geographical origin, especially in the case of *X. paraiberica* sp. nov., where each geographical population gave rise to a different haplotype, even more than one, as in the case of the Cantabria population (northern Spain). Nevertheless, the variability detected for these species was considerably lower than that detected in *X. macrodora* from the USA, where the variability found was up to 5%. Unfortunately, no information is available about other ribosomal markers for these American populations of *X. macrodora* [5, 10]. Molecular analyses of this study clearly supported the separation of the genera *Criconemoides* and *Xenocriconemella,* since well-separated lineages in the phylogenetic tree of ribosomal (28S rRNA) and mitochondrial (COI) loci were demonstrated (Figs. 4, 5, 6 and 7). In 28S rRNA, ITS region (except for JQ708139-*X. macrodora* from the USA, which need additional molecular confirmation with additional markers) and COI trees, the *Xenocriconemella* species complex clustered together in a single cluster (suggesting monophyly) and was clearly separated from *Criconemoides* spp., including *C. rosmarini*, which clustered in no resolved lineages with *Discocriconemella limitanea*, *Criconemoides annulatus* and *Lobocriconema iranense*. Our results on juvenile annuli ornamentation and molecular (ribosomal and mitochondrial loci) analysis showed that *Xenocriconemella* is a valid and separate genus from other genera within Criconematidae, and these results supported the hypothesis of several authors considering *Xenocriconemella* as a valid genus [2, 63]. In addition, since ornamented annuli are also a shared trait by a few species within the genus *Criconemoides*, including *C. ihlathum*, *C. lizarbus*, *C. ornativulvatus*, *C. silvicola*, and *C. tiaraensis* [2], further studies are needed to confirm the taxonomical status of these groups and their phylogenetic relationships.

This study gave molecular markers for the first time for *Criconemoides rosmarini,* two D2-D3 expansion segments of 28S rRNA and one partial COI sequence, which showed rather low similarity values with the accessions available in GenBank, with *Discocriconemella limitanea* being the most similar, with a value of 90%. Phylogenetic analyses based on the D2-D3, ITS, 18S and partial COI genes using BI resulted in a consistent position for the newly described species of the *Xenocriconemella* species complex from Spain, and *X. macrodora* from several geographical origins. The position of *C. rosmarini* within the D2-D3 and partial COI trees showed that the phylogenetic relationship with the species belonging to the *Xenocriconemella* species complex is quite distant. These results showed the monophyly of the genus *Xenocriconemella* by ribosomal and mitochondrial loci but also confirmed that the genus *Criconemoides* is polyphyletic, as already reported by other researchers [14].

## Conclusion

This study confirms that the globally distributed nominal *Xenocriconemella macrodora* species is a species complex composed of species that are morphometrically and morphologically similar, but clearly different at the molecular level. In this study, three new species (*X. iberica* sp. nov., *X. paraiberica* sp. nov., and *X. pradense* sp. nov.) are described by the application of integrative taxonomy. However, the molecular diversity of this species in USA and Italy confirmed that additional species are likely present in this species complex, and the diversity of this group may be higher than expected. The study of *X. macrodora* topotypes can clarify the position of this species using molecular markers under an integrative approach.

### Supplementary Information

**Additional file 1:** **Table S1.** Measurements of several populations of *Xenocriconemella macrodora* De Grisse and Loof, 1965 from several countries.

**Additional file 2:** **Fig. S1.** SEM micrographs of *Criconemoides rosmarini* (Castillo, Siddiqi and Gómez-Barcina, 1988) Siddiqi, 2000 female and juvenile. (a, b) female anterior region showing crenate annuli; (c, d) female lip region; (e, f) female en face view showing oral aperture; (g) female posterior region showing vulva and anus (arrowed); (h) juvenile tail. Abbreviations: a = anus; V = vulva. Scale bars: (a) = 10 µm; (b, h, g) = 5 µm; (d, e, f) = 1 µm; (c) = 2 µm.

## Data Availability

All data generated or analysed during this study are included in this published article. Sequences are deposited in GenBank, NCBI. This article has been registered at Zoobank (urn:lsid:zoobank.org:pub:B4A7D5EC-511D-4F75-A257-29745B56F363).
