# Supplementary figures and images for: The more we search, the more we find: discovering and expanding the biodiversity in the ring nematode genus Xenocriconemella De Grisse and Loof, 1965 (Nematoda: Criconematidae)
Source: Zoological Lett. 2024 Mar 25;10:8. doi: 10.1186/s40851-024-00230-3 (PMC10962084; doi:10.1186/s40851-024-00230-3)

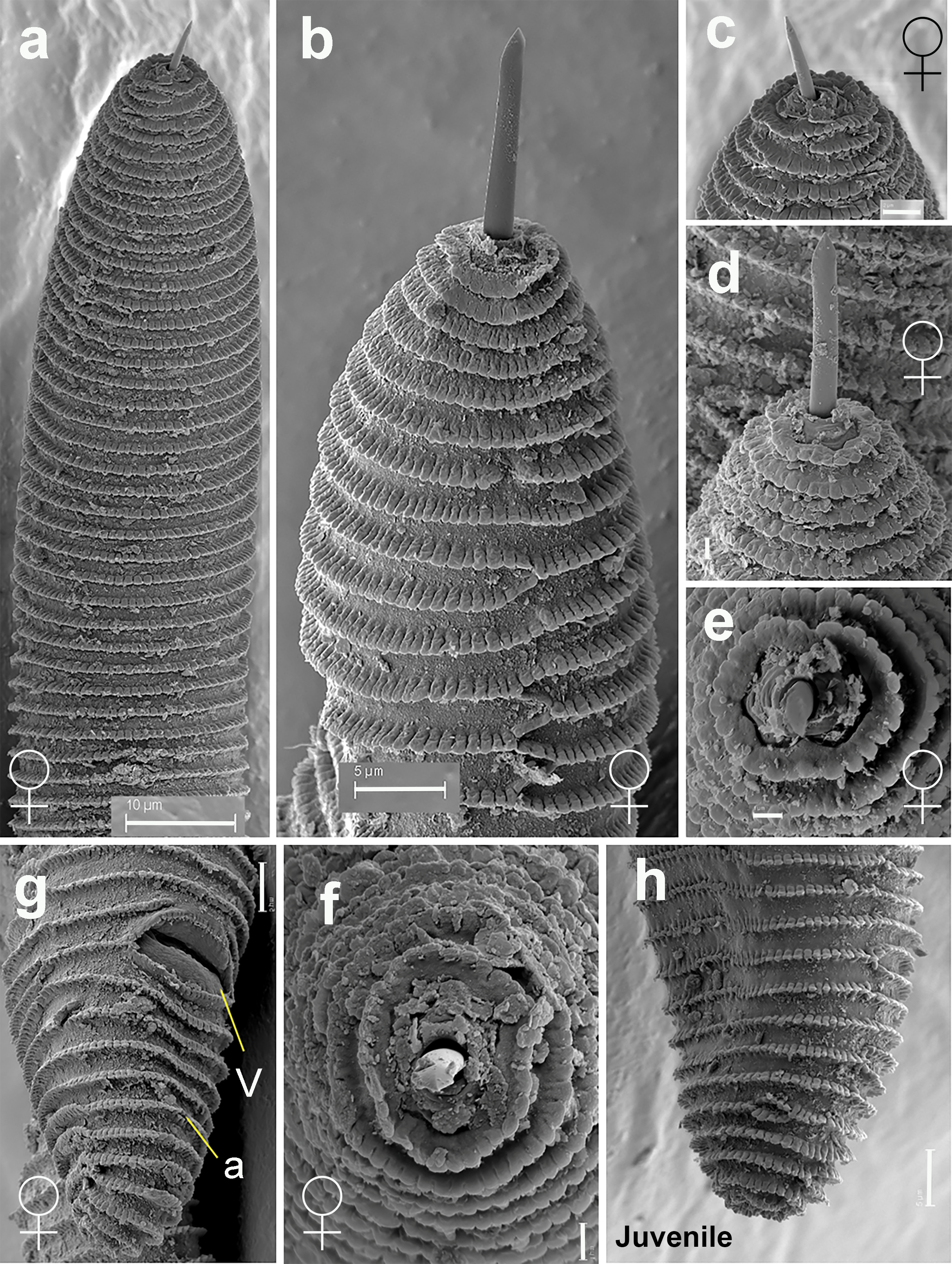

Supplement: Supplementary file 2 — Additional file 2: Fig. S1. SEM micrographs of Criconemoides rosmarini (Castillo, Siddiqi and Gómez-Barcina, 1988) Siddiqi, 2000 female and juvenile. (a, b) female anterior region showing crenate annuli; (c, d) female lip region; (e, f) female en face view showing oral aperture; (g) female posterior region showing vulva and anus (arrowed); (h) juvenile tail. Abbreviations: a = anus; V = vulva. Scale bars: (a) = 10 µm; (b, h, g) = 5 µm; (d, e, f) = 1 µm; (c) = 2 µm. [file 40851_2024_230_MOESM2_ESM.tif]
